# The phase-seeding method for solving non-centrosymmetric crystal structures: a challenge for artificial intelligence

**DOI:** 10.1107/S2053273325002797

**Published:** 2025-04-17

**Authors:** Benedetta Carrozzini, Liberato De Caro, Cinzia Giannini, Angela Altomare, Rocco Caliandro

**Affiliations:** a Institute of Crystallography National Research Council of Italy via Amendola 122/o Bari 70126 Italy; Czech Academy of Sciences, Czechia

**Keywords:** crystal structure solution, crystallographic methods, phase seeding, artificial intelligence

## Abstract

The overall crystallographic process involves acquiring experimental data and using crystallographic software to find the structure solution. Unfortunately, while diffracted intensities can be measured, the corresponding phases – needed to determine atomic positions – remain experimentally inaccessible (phase problem). Direct methods and the Patterson approach have been successful in solving crystal structures but face limitations with large structures or low-resolution data. Current artificial intelligence (AI) based approaches, such as those recently developed by Larsen *et al.* [*Science* (2024), **385**, 522–528], have been applied with success to solve centrosymmetric structures, where the phase is binary (0 or π). The current work proposes a new phasing method designed for AI integration, applicable also to non-centrosymmetric structures, where the phase is a continuous variable. The approach involves discretizing the initial phase values for non-centrosymmetric structures into a few distinct values (*e.g.* values corresponding to the four quadrants). This reduces the complex phase problem from a continuous regression task to a multi-class classification problem, where only a few phase seed values need to be determined. This discretization allows the use of a smaller training dataset for deep learning models, reducing computational complexity. Our feasibility study results show that this method can effectively solve small, medium and large structures, with the minimum percentage of phase seeds (three or four points in the interval [0, 2π]), and 10% to 30% of seed symmetry-independent reflections. This phase-seeding method has the potential to extend AI-based approaches to solve crystal structures *ab initio*, regardless of complexity or symmetry, by combining AI classification algorithms with classical phasing procedures.

## Abstract 2

A new phasing method capable of solving crystal structures, from small molecules to macromolecules, is conceived and tested by a feasibility study.

## 1. Abbreviations

AI: artificial intelligence.

MPE: mean phase error.

*Rf*: crystallographic agreement factor.

*N*_asym_: number of non-hydrogen atoms in the asymmetric unit.

*N*_refl_: number of symmetry-independent reflections.

EDM: electron-density modification.

φ_a_: actual phase value.

φ_d_: discretized phase value.

Perc_seed_: percentage of the number of seed reflections with respect to *N*_refl_.

Perc_lim_: minimum Perc_seed_ for which the phasing procedure leads to the correct structure solution.

MPE_lim_: maximum MPE for which the phasing procedure leads to the correct structure solution.

*E*: reflection-normalized structure-factor amplitude.

## 2. Introduction

For almost a century, the solution at the atomic level of unknown crystal structures has been the main aim of crystallography. Experiments offer the possibility to measure only diffracted intensities from crystals, with a Bragg discrete sampling. Bragg intensities, sampled on the reciprocal-space nodes, are related to the Fourier transform of the electron density of the unknown structures. However, any information about the corresponding phases, which is essential to derive atomic positions within the crystal unit cell through a Fourier synthesis of the square root of the scattered intensities, is missing (crystallographic phase problem). Direct methods (DM) (Karle & Hauptman, 1950 ▸) revolutionized crystallography by using probabilistic approaches to solve the phase problem. DM rely on relationships among the phases of different reflections and have been particularly successful for small and medium-sized molecules (Giacovazzo, 2014 ▸). The theory requires that atoms are completely resolved as separate objects (Sayre, 1952 ▸). If this condition does not hold, then the probabilistic principle, on which DM depend, loses significant efficiency to reliably estimate unknown phases. DM are typically used successfully to solve the phase problem for small and medium-sized molecules.

The alternative Patterson approaches (Patterson, 1934 ▸) use direct-space maps to determine interatomic distances directly from the inverse Fourier transform of diffraction intensities. These *ab initio* approaches are particularly effective even for large-size structures, when heavy atoms are present, through suitable deconvolution procedures of the Patterson maps (Burla *et al.*, 2006 ▸).

Moreover, atomic resolution is often not available in experimental data. Extrapolation methods involve mathematical techniques to predict the reflection intensities that are not directly observed, allowing for the extension of diffraction data to higher resolution. This method has been useful in improving the accuracy of phase determination (Caliandro *et al.*, 2005*a* ▸,*b* ▸).

*Ab initio* approaches (dual-space methods), combined with EDM techniques (Zhang *et al.*, 2006 ▸), are highly effective for determining macromolecular structures when (quasi-)atomic resolution data are available (Weeks *et al.*, 1994 ▸; Schneider & Sheldrick, 2002 ▸; Jia-xing *et al.*, 2005 ▸; Burla *et al.*, 2015 ▸). They can also be used to locate the substructure of heavy atoms or anomalous scatterers in SIR–MIR (single/multiple isomorphous replacement) or SAD–MAD (single-/multiple-wavelength anomalous diffraction) experiments.

Remarkable developments in *ab initio* methods, supported by their implementation in advanced software, running on high-performance computers, have greatly simplified and automated the structure solution of crystalline compounds with varying chemical compositions and complex structures, and made a substantial impact on a wide range of scientific fields.

This progress has shifted focus towards areas where solving structures presents significant challenges, such as proteins or microcrystalline powder structures.

Since the 1990s, several methods have been developed to tackle the problem of crystal structure solution from powder diffraction data. However, even for small-sized structures, solving powder structures remains challenging in many cases, as powder diffraction data often contain uncertainties intrinsically arising from the experimental process. Consequently, in powder diffraction, not only the phases are unknown, but the reliability of the diffraction intensities is also low (Altomare *et al.*, 2019 ▸), further declining at high resolution due to peak overlapping, a common challenge in this technique. These factors complicate the use of DM in the solution process. For powder diffraction, direct-space methods have greatly improved the crystal structure determination by avoiding the limitations of DM and effectively handling low-resolution data. Direct-space methods involve generating trial crystal structures within the crystal unit cell, with each trial’s reliability assessed by comparing the calculated diffraction pattern with the experimental data. The approach models the observed pattern as a whole, optimizing the structural model to achieve the best data fit (David, 2019 ▸; Černý & Favre-Nicolin, 2019 ▸; Shankland, 2019 ▸; Cuocci *et al.*, 2022 ▸). However, the initial structural knowledge used to generate the trial structures must be reliable and accurately represent the system under investigation, particularly in terms of bond distances and angles. When this condition is not met, direct-space methods can be unsuccessful.

For homologous proteins, the phase problem is frequently addressed using molecular replacement (MR). This technique involves the rigid-body placement (both the orientation and position) of a search model – an identical or structurally similar protein – within the asymmetric unit of a target crystal to minimize the root mean square deviation between the two structures. The best configuration is determined by the agreement between calculated and observed structure factors, assessed using various MR search functions, as implemented in different software (*e.g.* Fujinaga & Read, 1987 ▸; Navaza, 1994 ▸; Glykos & Kokkinidis, 2000 ▸; Read, 2001 ▸; McCoy *et al.*, 2007 ▸; Caliandro *et al.*, 2009*b* ▸; Vagin & Teplyakov, 2010 ▸). MR has been instrumental in solving the structures of many proteins, especially when high-quality homologous models are available (Rossmann, 2001 ▸).

AI, particularly through tools like *AlphaFold*, has revolutionized the prediction of protein structures from amino acid sequences. *AlphaFold*, developed by DeepMind, uses deep learning techniques to predict the 3D structure of proteins with remarkable accuracy. This AI-driven approach has significantly advanced our ability to model protein structures, even in cases where experimental data are limited (Jumper *et al.*, 2021 ▸). However, there are still many unknowns, especially for novel protein folds that lack homologous structures. These new folds often require innovative approaches to accurately determine their phases and structures (Vila, 2023 ▸).

To highlight the relevance of AI tools in protein structure prediction, the 2024 Chemistry Nobel Prize was awarded to J. Jumper and D. Hassabis for developing *AlphaFold*, along with D. Baker for advancements in computational protein design, which have been strengthened by AI in recent years.

Nanomaterials present unique challenges for phase determination due to their small size and often complex structures. Traditional crystallographic methods may be insufficient to resolve phases accurately. New techniques, such as phase engineering and the use of advanced microscopy, are being explored to address these challenges (Shi *et al.*, 2024 ▸). These unresolved issues highlight the ongoing need for innovation and development in the field of crystallography to improve phase determination and structural analysis.

The role of AI, in particular machine learning (ML), in crystallography has been expanding rapidly, impacting various fields (Billinge & Proffen, 2024 ▸; Greasley & Hosein, 2023 ▸; Nawaz *et al.*, 2023 ▸; Surdu & Győrgy, 2023 ▸; Guccione *et al.*, 2023 ▸). Recent advances in developing neural networks trained on large datasets show promise in providing more accurate phase predictions, even from low-resolution data, searching for the solution in both the reciprocal space (Larsen *et al.*, 2024 ▸) and the direct space (Pan *et al.*, 2023 ▸).

Larsen *et al.* (2024 ▸) show that using reciprocal space is better than direct space (Pan *et al.*, 2023 ▸), since the variables to be determined in the direct space, *i.e.* the positions of atoms, vary continuously within the crystalline cell. The limitations of this novel approach (Larsen *et al.*, 2024 ▸) are the limit on the maximum unit-cell dimensions smaller than 1 nm and the restriction to centrosymmetric structures which, in turn, limits the phase to a binary variable (0 or π). These modest limits are imposed by the computation costs of implementing an efficient deep learning neural network. Therefore, extending AI approaches to non-centrosymmetric structures is challenging, because the phase, which, in general, is a continuous variable, complicates the application of multi-class approaches. These approaches typically rely on a limited set of possible phase values, which significantly reduces computational costs and enhances efficiency. However, this constraint seems to prevent their direct use in handling the continuous nature of the phase in non-centrosymmetric structures.

To overcome these limitations, in this work we propose a novel phasing method, tailored for integration with AI techniques. In the first step of the phasing process, initial guess phases for non-centrosymmetric structures are discretized into a few discrete values, such as those corresponding to the four quadrants’ values. This strategy could allow reduction of the mathematical crystallographic phase problem from a general statistical regression problem, needed for retrieving the values of a continuous phase variable, to a multi-class classification problem, in which only very few values must be determined, in analogy with the 0 and π values for centrosymmetric structures. Moreover, the reduction of the general phase problem (continuous variable problem) to a multi-class classification problem (discrete variable problem) could allow the use of a less extended input set for the training step of deep learning networks. In fact, assigning random initial phase values for a centrosymmetric structure, as demonstrated by Larsen *et al.* (2024 ▸), effectively sets the correct values for half of the reflections. In this case, the phase acts as a binary variable, which reduces the AI task to determining the correct values for the other half of the reflections.

Therefore, to mitigate the high computational costs associated with implementing AI techniques for solving the phase problem in arbitrary crystallographic space groups, this work presents a proof of concept aimed at approximating initial continuous phase values for non-centrosymmetric structures by using discrete values from four quadrants (0, π/2, π, 3π/4) or other discrete approximations, such as (0, π), (0, 2π/3, 4π/3) or (0, π/3, 2π/3, π, 4π/3, 5π/3).

## 3. Methods

**Figure 1:**
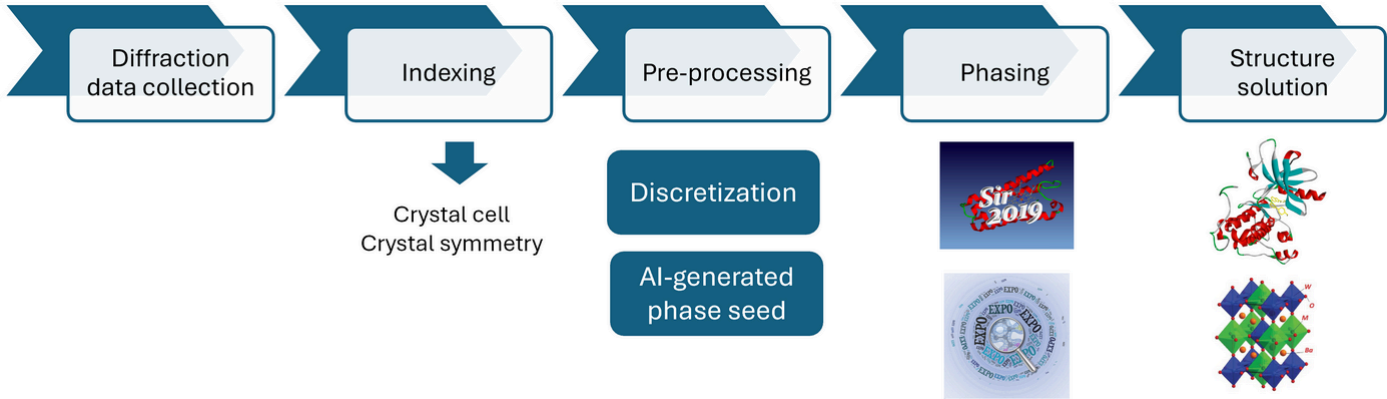
Scheme of the phase-seeding procedure used for crystal structure determination.

We propose here a general procedure to solve non-centrosymmetric crystal structures, based on the concept of phase seeding and the use of AI. The steps involved in the procedure are outlined in Fig. 1 ▸. Experimental data acquired in single-crystal or powder X-ray diffraction experiments are preliminarily subjected to indexing procedures to determine the crystal cell parameters and symmetry. This approach uniquely defines the reciprocal lattice and allows for the identification of reflections with restricted phase values, if present, as well as those with general phase values. Each node of the reciprocal lattice corresponds to a Bragg reflection, which is characterized by a diffraction intensity. This intensity is directly measured in the case of single-crystal X-ray diffraction experiments or obtained by extracting intensities from an X-ray powder diffraction pattern. Additionally, each reflection has a corresponding phase value. The obtained reflection set is then submitted to a pre-processing step where all possible phase values, ranging continuously from 0° to 360° for general reflections of non-centrosymmetric structures, are discretized by sampling a few points (from 2 to 6) within this range. In addition, a set of discrete phase values is generated for restricted phase reflections, if they are expected by the symmetry. In this paper, the task of the phase seed generation is carried out by an automatic procedure, but it can also be effectively performed using a typical AI process. Experimental amplitudes and calculated phases are used as input for a phasing procedure within crystallographic software, which typically operates iteratively in both direct and reciprocal space, implementing phase extension and refinement. At the end of the phasing procedure the electron-density map is calculated in direct space, and it is interpreted in terms of an atomistic model by automatic model-building computational routines. The crystal structures so obtained can then be validated against experimental data and stereochemistry restraints. This general architecture can in principle be applied to small molecules and biological macromolecules, provided the AI is trained on each specific type of structure.

### 3.1. Discretization

**Figure 2:**
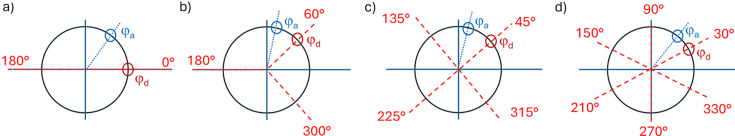
Discretization of phase values, according to four different sampling hypotheses: 2 values (*a*), 3 values (*b*), 4 values (*c*) and 6 values (*d*). The actual phase value (φ_a_, blue circle) and the corresponding discretized one (φ_d_, red circle) are highlighted for each sampling case.

Non-centrosymmetric space groups have most of the reflections with phase values ranging in a continuous way from 0 to 360° and, for some space groups, a small number of special reflections with restricted phase values, which can assume two values separated by 180°. The percentage of special reflections, relative to general ones, is determined by the crystal symmetry. To reduce the complexity of the problem, the phase values of general reflections have been discretized by four different types of sampling density, as shown in Fig. 2 ▸. Given the actual value φ_a_ of the phase of each general reflection, the corresponding discretized value φ_d_ is assumed as the phase of the nearest sampling point along the circle of unit radius. For restricted phase reflections, φ_a_ can assume only two values 180° apart and the corresponding φ_d_ is randomly chosen among these allowed values. For example, in the case of restricted phases of 0° and 180°, φ_d_ is assigned according to the first sampling density shown in Fig. 2 ▸(*a*) and limited to one of these two allowed phase values.

### 3.2. Phase seed generation

Random phase values are initially assigned to all input reflections. Given these random values φ_a_, the corresponding discretized values φ_d_ for general reflections are then determined according to the selected sampling density (Fig. 2 ▸). For special reflections, φ_d_ is restricted to the allowed discrete values. A subset of seed reflections is chosen randomly among the set of experimental symmetry-independent reflections, including both general and phase-restricted reflections. The percentage of seed reflections with respect to the total number of independent reflections, Perc_seed_, is defined and varied. For seed reflections, φ_a_ is given by the true phase value calculated from the published crystal structure, and φ_d_ is associated by considering the nearest sampling value of a general reflection. For restricted phase reflections, seed phase values coincide with the true values, since φ_a_ and φ_d_ are equal. Different ways of generating the phase seed have been explored and are compared in Section 4.6, where selections of seed reflections, based on data resolution or intensity, have been attempted and applied on a set of crystal structures.

### 3.3. Phase extension and refinement

**Figure 3:**
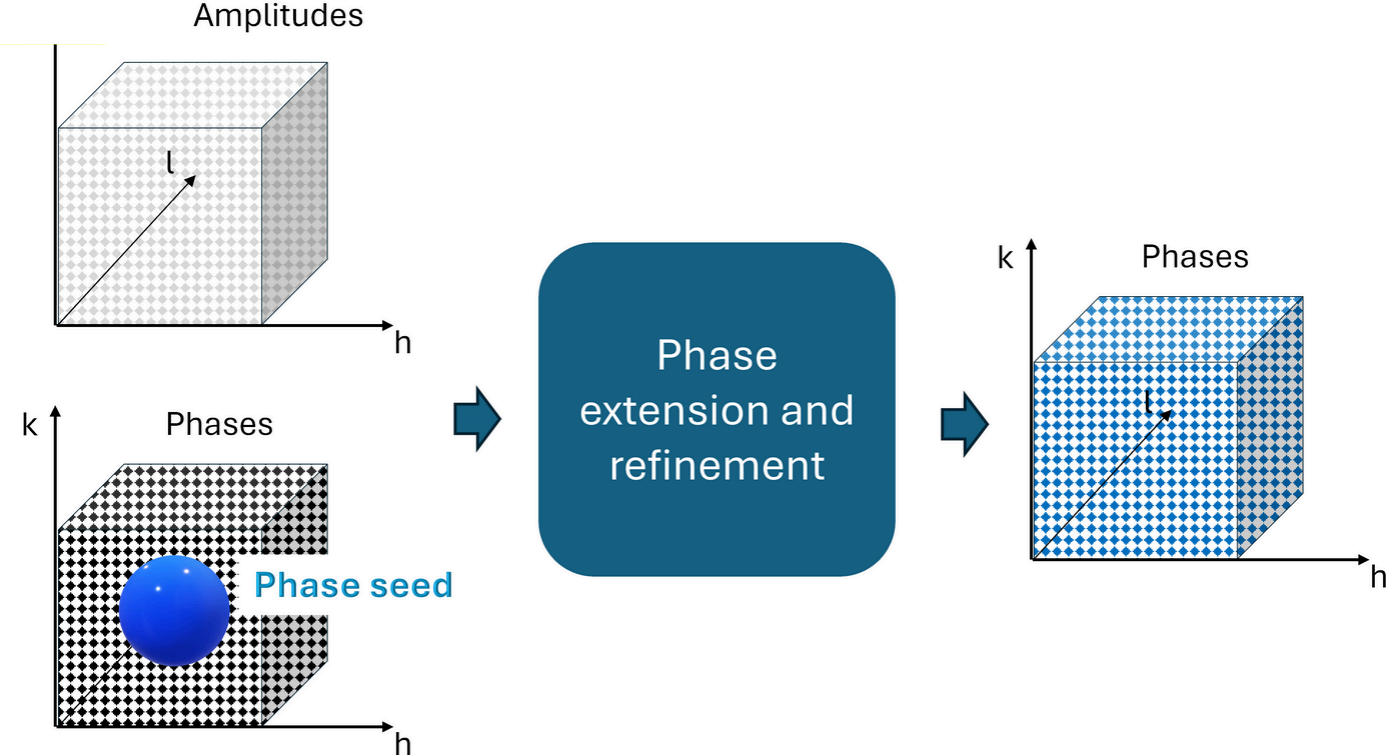
Scheme of the procedure to process X-ray diffraction data to produce a full set of phases. Reflections holding phase values close to the true ones are shown in blue.

Measured amplitudes and phase values assigned as explained in Section 3.2 are used as input for standard phasing procedures. As shown in Fig. 3 ▸, the initial phase values are chosen randomly for most reflections and set equal to their true values only for a small number (seed) of reflections. For non-seed reflections, the initial phase values are discretized according to one of the hypotheses shown in Fig. 2 ▸. The aim of the phasing procedure is both to propagate the good phase information from the seed of reflections to all the symmetry-independent reflections (phase extension), and to improve the phase values going from discrete values to continuous values (phase refinement). The phase extension and refinement procedure operates EDM cycles, where measured amplitudes are used as experimental constraints. Electron-density-map modifications are applied in direct space to enforce the atomistic hypothesis and propagated in reciprocal space as new phase values assigned to measured reflections (Cowtan, 1994 ▸; Giacovazzo & Siliqi, 1997 ▸; Burla *et al.*, 2005 ▸). Non-measured reflections are also considered in the recycling, according to the ‘free lunch’ procedure (Caliandro *et al.*, 2007 ▸). They have the effect of avoiding the phase refinement being trapped in local minima, and artificially increasing the data resolution and quality of the electron-density map (Caliandro *et al.*, 2008 ▸). In the case of protein structures, molecular envelope calculations are added to the EDM to describe regions delegated to the solvent (Burla *et al.*, 2003 ▸). Data from protein crystals collected at low resolution (>2.0 Å) are treated by the *Synergy-CAB* pipeline (Carrozzini *et al.*, 2023 ▸), which includes EDM cycles based on the difference electron-density map (Caliandro *et al.*, 2009*a* ▸; Burla *et al.*, 2011 ▸), coupled with automated model building (Cowtan, 2006 ▸, 2014 ▸; Langer *et al.*, 2008 ▸; Terwilliger *et al.*, 2008 ▸) and refinement procedures (Murshudov *et al.*, 2011 ▸). After the phase extension and refinement procedure, a new phase value is assigned to all symmetry-independent reflections, with the result that the good phase values, initially confined to the seed reflections, are propagated to all the reflections.

The quality of the final phase values is checked by calculating the mean phase error (MPE) relative to the phase values derived from the known structural model, as well as by determining the crystallographic agreement factor (*Rf*) between the calculated and observed amplitudes. Based on these two figures of merit, which operate in reciprocal and direct space, respectively, we can evaluate the validity of the crystal structure solution and assess the efficiency of the phase seed generation.

### 3.4. Computer programs

The *SIR2014* software (Burla *et al.*, 2015 ▸) was used to generate the phase seed, calculate the MPE as a function of the phase seed size (*i.e.* the percentage of reflections assigned with the correct discretized phase values, Perc_seed_) and carry out the phasing extension and refinement procedure. This latter task is accomplished in different ways depending on the type of structure to be solved. For microcrystalline structures, the program *EXPO2014* (Altomare *et al.*, 2013 ▸) was used to extract the reflection intensities from the experimental powder diffraction patterns of a few published powder structures, whose cell parameters and space groups had been determined. The extracted reflection intensities were then treated as single-crystal data and used as input to *SIR2014*.

### 3.5. Structure selection

The phase-seeding procedure was tested on 100 known crystal structures taken from the Crystallographic Open Database (COD) (Gražulis *et al.*, 2009 ▸), the Cambridge Structural Database (CSD) (Groom *et al.*, 2016 ▸) and the Protein Data Bank (PDB) (Berman *et al.*, 2000 ▸), whose crystallographic properties are listed in Tables S1, S2 and S3 in the supporting information. The structures are categorized into small (*N*_asym_ < 80), medium (80 < *N*_asym_ < 300) and large (*N*_asym_ > 300) structures, depending on the number of non-hydrogen atoms in the asymmetric unit. Six structures having 200 < *N*_asym_ < 300 have been included in the set of large structures, since they are biological macromolecules taken from the PDB. For each group of structures, relevant variables affecting the phasing procedures, such as the presence of heavy atoms, crystal symmetry, data resolution and unit-cell dimensions, were evenly sampled to effectively test the phasing seed procedure across a wide range of cases. It should be noted that the set of test structures includes two structures that do not have symmetry-restricted reflections, *i.e.* the small structure with COD code 2218160, with space group *R*3 (Table S1), and the large structure with PDB code 1gyo, space group *P*3_1_ (Table S3). Four structures solved by X-ray powder diffraction data are also included in the test set (Table S4).

## 4. Results

The results are reported separately, dividing the test structures according to their size, *i.e.* the number of non-hydrogen atoms in the asymmetric unit. The results address both the characterization of the pre-processing step, *i.e.* the evaluation of the MPE of the pre-processed dataset obtained by using phase values assigned with different sampling densities and phase seeds with different size (Perc_seed_), and the phasing step, *i.e.* the assessment of the minimum size of the phase seed that leads to a valid structure solution (Perc_lim_).

### 4.1. Small structures

**Figure 4:**
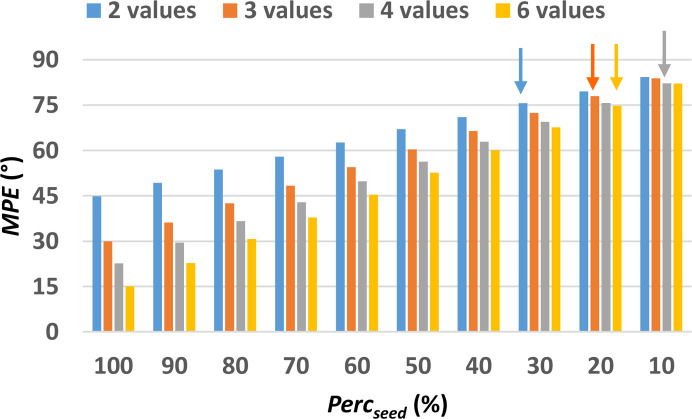
MPE of the discretized phase values with respect to the true values as a function of the size of the phase seed, *i.e.* the percentage of reflections assigned with the correct discretized phase values (Perc_seed_). The minimum Perc_seed_ for each one of the 4 different sampling densities, for which the initial set of phases converge towards a solution (Perc_lim_), are highlighted by arrows. Calculations have been performed on the small test structure COD 2225745, the first of those listed in Table S1.

The evaluation of the MPE obtained after the pre-processing step for the non-centrosymmetric test structure with COD number 2225745 (Table S1) is shown in Fig. 4 ▸. Here MPE is plotted as a function of the size of the phase seed, measured by Perc_seed_, and the sampling density used. It can be noted that even when only 2 values are used to define the phase value of general reflections, MPE remains below 50° for Perc_seed_ > 80%, and with even lower values obtained for higher sampling densities. It is worth noting that the difference in MPE values at various sampling densities is most evident for larger seed size. When instead Perc_seed_ is below 30% phase sampling with 2 or 6 values is almost equivalent.

The assessment of the phasing procedure has been carried out by considering the sampling densities from 2 to 6 and all the considered Perc_seed_ values. For this structure, the Perc_lim_ value is 30% for sampling with 2 angles, 20% for sampling with 3 and 10% for sampling with 4 angles, and again 20% for sampling with 6 values. The corresponding MPE_lim_ values are 75.6°, 77.9, 82.1° and 74.7°, respectively (see arrows in Fig. 4 ▸). It is interesting to note that increasing the sampling density from 4 to 6 worsens the phasing results, as a seed with larger size (Perc_seed_ 20% instead of 10%) and smaller MPE (MPE_lim_ 74.7° instead of 82.1°) are needed to converge to the solution.

This structure is easily solved by the modern direct methods (MDM) procedure implemented in *SIR2014*. The first trial of the tangent procedure (Burla *et al.*, 2015 ▸) is able to phase 10% of the *N*_refl_ reflections with higher *E* values with MPE = 20°, and subsequent EDM cycles extend this phase information reaching MPE = 10° over the *N*_refl_ reflections.

**Figure 5:**
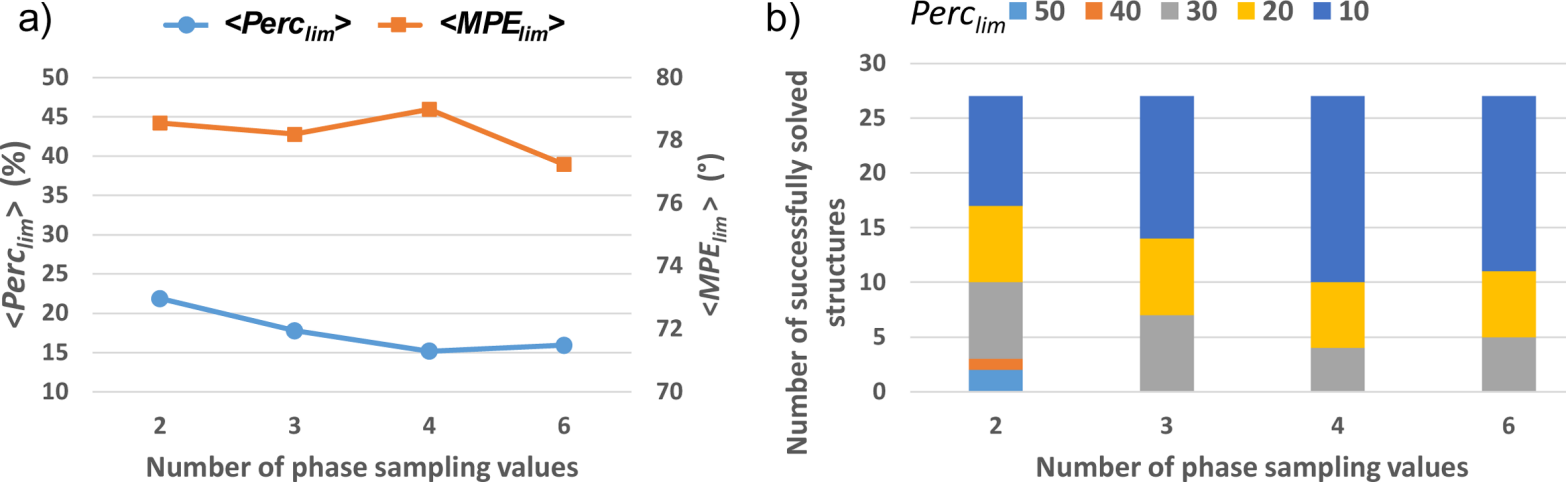
Results of the phase-seeding procedure on the full set of small test structures used in this study. (*a*) Perc_lim_ (left axis) and MPE_lim_ (right axis) and (*b*) the number of successfully solved test structures plotted for Perc_lim_ values (%) ranging from 10 to 50 as a function of the sampling density.

The results of the phasing procedure obtained for the full set of small test structures are shown in Fig. 5 ▸. The efficiency of the phasing procedure as a function of the sampling density follows an expected increasing trend, with both Perc_lim_ and MPE_lim_ decreasing when a larger number of sampling values is used [Fig. 5 ▸(*a*)]. However, deviations from this trend frequently occur among the test structures, as seen in Fig. 4 ▸ for the structure with COD number 2225745 relative to the sampling density with 4 values. These deviations are responsible for the anomaly in the MPE_lim_ curve in Fig. 5 ▸(*a*), which corresponds to a higher value obtained when sampling with 4 points. This could be due to a greater ease of exploring phase space when sampling at lower density, preventing the phasing procedure from getting trapped in local minima. As a matter of fact, the highest number of test structures successfully solved with the minimum seed size (Perc_seed_ = 10) is obtained when sampling with 4 points [Fig. 5 ▸(*b*)].

An overall study through the entire set of small test structures is reported in the supporting information (Section S3). For the pre-processing step, Fig. S2 shows that the MPE values obtained after pre-processing depend linearly on the size of the phase seed, and the slope of this dependence is nearly constant throughout the test structures, despite the fact that it depends on the number of sampling points, as already seen in Fig. 4 ▸. From Fig. S2 it can be noted that when only 2 sampling points are used, the MPE obtained for maximum seed size, *i.e.* when Perc_lim_ = 100, has a large variability with respect to the cases in which more sampling points are used. The lower MPE values are reached for crystal structures with higher symmetry, and in fact the MPE values anti-correlate with the number of symmetry operators, as shown in Fig. S3. This trend reduces for higher sampling densities, so it can be attributed to an artefact due to the poor sampling of the phase values.

For the phasing step, Figs. S4(*a*), S4(*b*) show that the Perc_lim_ values are between 10% and 30% and they have a positive correlation with data resolution, and a negative correlation with the atomic number of the heaviest atom in the crystal cell (*Z*_max_). The corresponding MPE_lim_ values [Figs. S4(*c*), S4(*d*)] range between 65° and 85° and, as expected, exhibit opposite trends to Perc_lim_ when correlated with data resolution and *Z*_max_. The correlation with other crystallographic variables is less significant.

### 4.2. Medium structures

**Figure 6:**
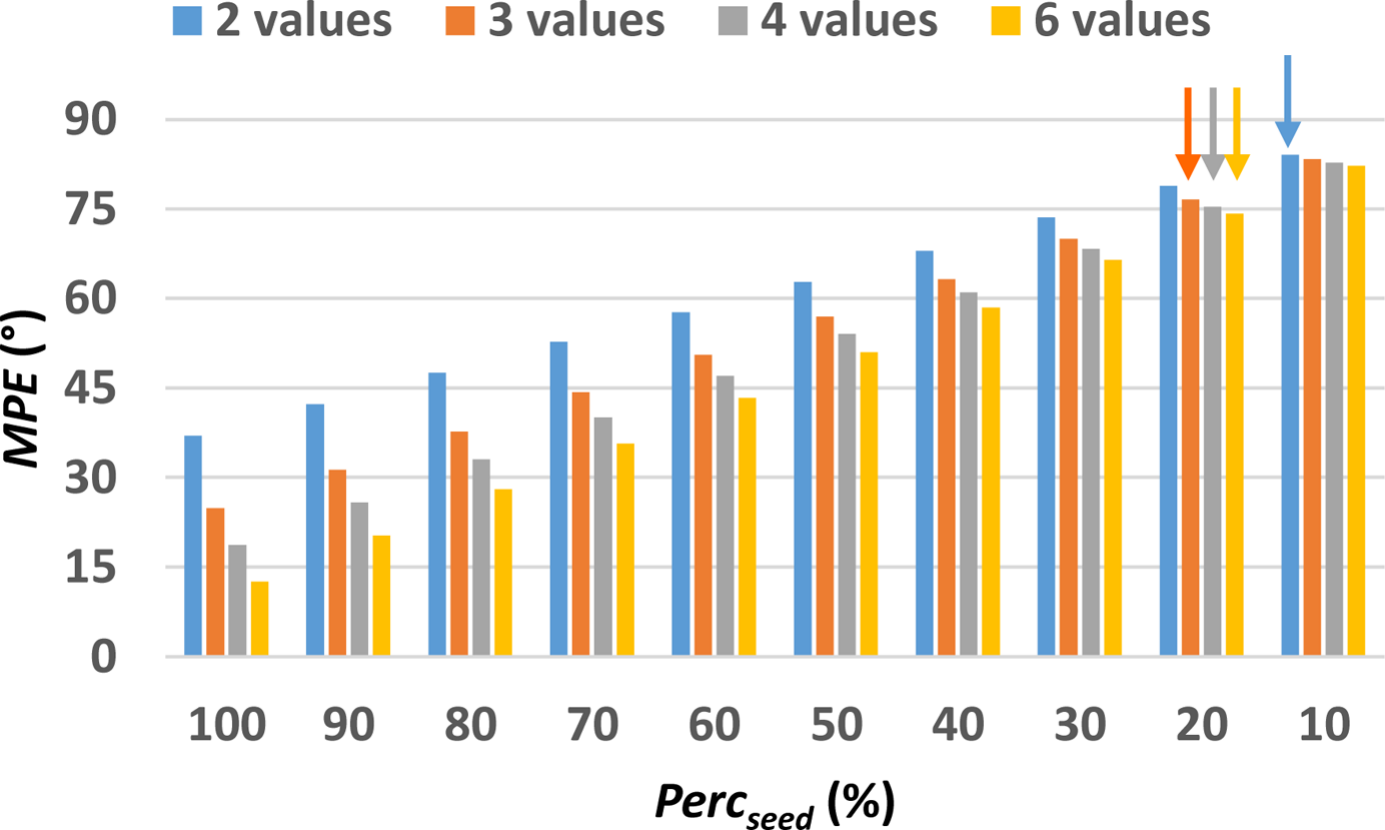
MPE of the discretized phase values with respect to the true values as a function of the size of the phase seed, measured by Perc_seed_, *i.e.* the percentage of reflections to which the true discretized phase values have been assigned. The lowest Perc_seed_ for which the initial set of phases converged towards a solution are highlighted by arrows. Calculations have been performed on the medium test structure with COD 2012193, the first of those listed in Table S2.

The results of the pre-processing step applied to the medium-sized structure with COD number 2012193 (see first row in Table S2) are shown in Fig. 6 ▸. Besides the overall similarity with results obtained for the small test structure (Fig. 4 ▸), medium-sized structures exhibit a more marked decrease of the MPE as a function of Perc_seed_ than the small-sized structures. This is confirmed by the overall analysis on all the structures listed in Table S2 (Fig. S5). Even for this structure, the result of the phasing process is surprisingly different from what we expected, given that the smaller Perc_lim_ is obtained by the coarser sampling (2 values). Higher sampling densities produce worse results, *i.e.* higher Perc_lim_. This structure can be solved *ab initio* by using the MDM procedure (Burla *et al.*, 2015 ▸) in *SIR2014*. The structure solution is reached only at the 46th trial, and after using the RELAX procedure (Burla *et al.*, 2000 ▸, 2002 ▸).

**Figure 7:**
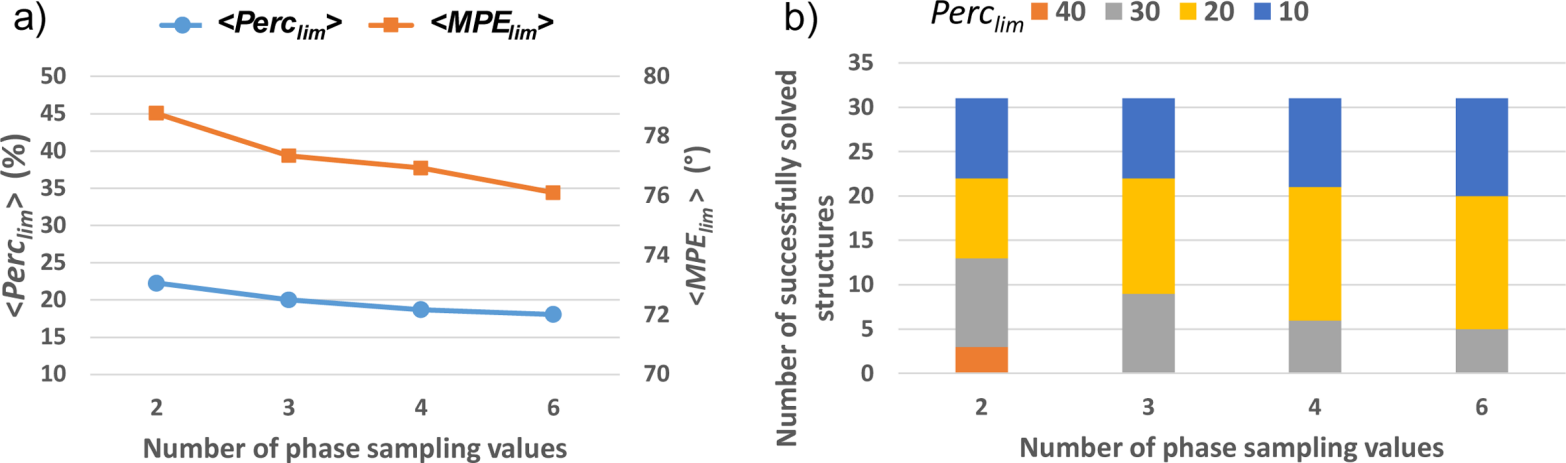
Results of the phase-seeding procedure on the full set of medium test structures used in this study. (*a*) Perc_lim_ (left axis) and MPE_lim_ (right axis) and (*b*) the number of successfully solved test structures plotted for Perc_lim_ values (%) ranging from 10 to 50 as a function of the sampling density.

Despite the anomaly found for the first structure in Table S2, when the averages among all the medium structures are considered, the trends of Perc_lim_ and MPE_lim_ as a function of the sampling density are both decreasing, as expected [Fig. 7 ▸(*a*)]. Most of the medium test structures are solved with Perc_lim_ = 20, whereas small structures were mostly solved with Perc_seed_ = 10 [*cf*. Fig. 7 ▸(*b*) and Fig. 5 ▸(*b*)], and the distribution of Perc_lim_ is nearly similar when using 4 and 6 phase values. [Fig. 7 ▸(*b*)].

The overall results of the pre-processing step for medium structures (shown in Section S4) confirm what has already been observed for small structures.

As regards the pre-processing step, the MPE values decrease linearly as Perc_seed_ increases, with a slope that is constant among the test structures but increases with the number of sampling points (Fig. S5). Moreover, the MPE values anti-correlate with the number of symmetry operators, with a dependence increasing at lower sampling densities (Fig. S6).

Regarding the phasing step, Fig. S7 shows that the Perc_lim_ values are between 10% and 30% even for medium structures, they have a positive correlation with data resolution, and a negative correlation with *Z*_max_ [Figs. S7(*a*), S7(*b*)]. The corresponding MPE_lim_ values are still between 65° and 85° [Figs. S7(*c*), S7(*d*)].

### 4.3. Large structures

**Figure 8:**
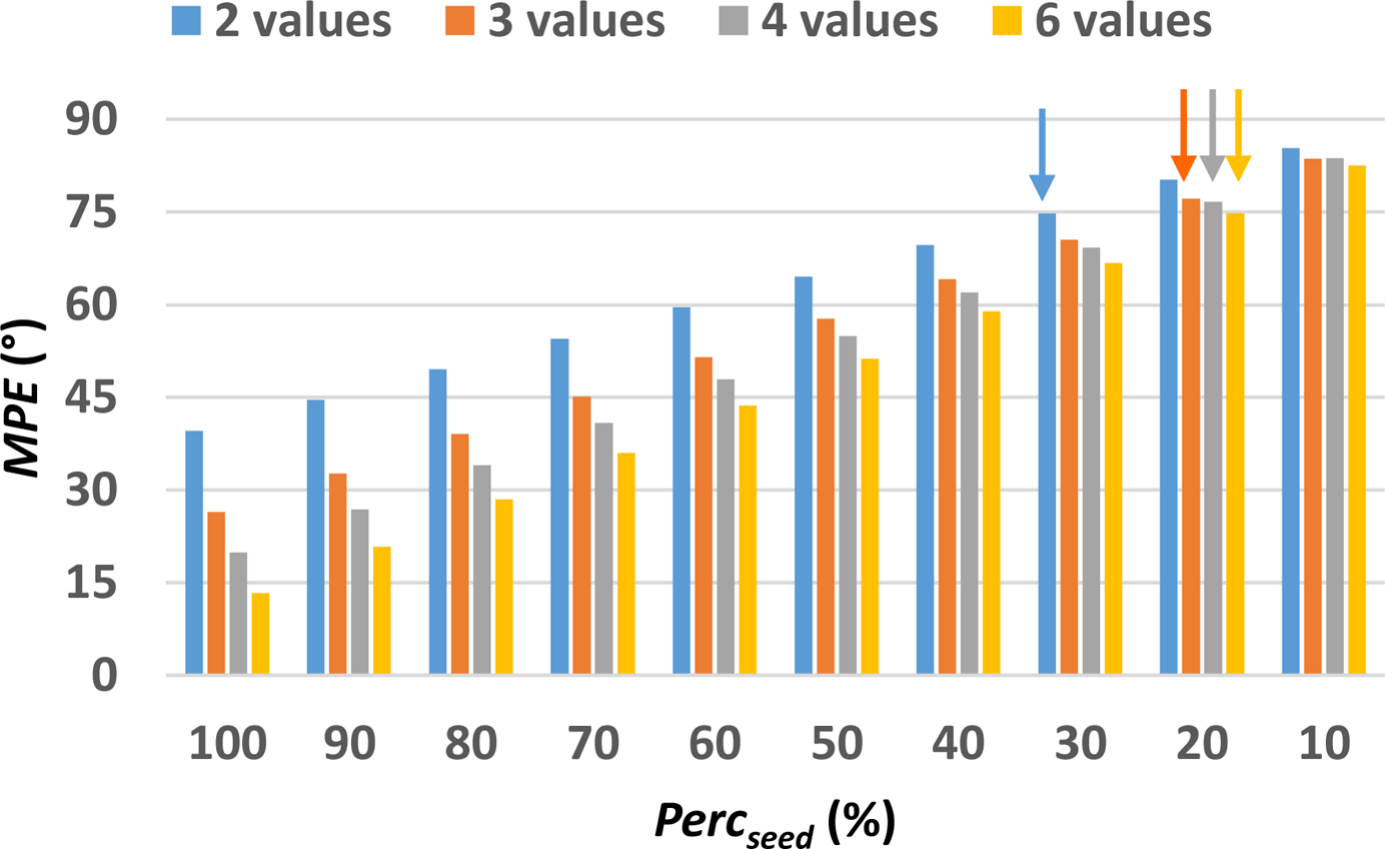
MPE of the discretized phase values with respect to the true values as a function of the size of the phase seed, measured by Perc_seed_, *i.e.* the percentage of reflections to which the true discretized phase values have been assigned. The lowest Perc_seed_ for which the initial set of phases converged towards a solution are highlighted by arrows. Calculations have been performed on the large test structure PDB 193l, the first of those listed in Table S3.

An example of application of the pre-processing step to a large structure (the protein with PDB code 193l, whose crystallographic data are shown in the first row of Table S3) is shown in Fig. 8 ▸. The MPE values and their dependence on Perc_seed_ are similar to those seen for medium structures (Fig. 6 ▸). For the 193l structure, the results of the phasing process are in line with what is expected, as the coarser phase sampling with 2 values performs worse (Perc_lim_ = 20) with respect to higher sampling densities (Perc_lim_ = 10). This structure cannot be solved *ab initio* by *SIR2014*.

**Figure 9:**
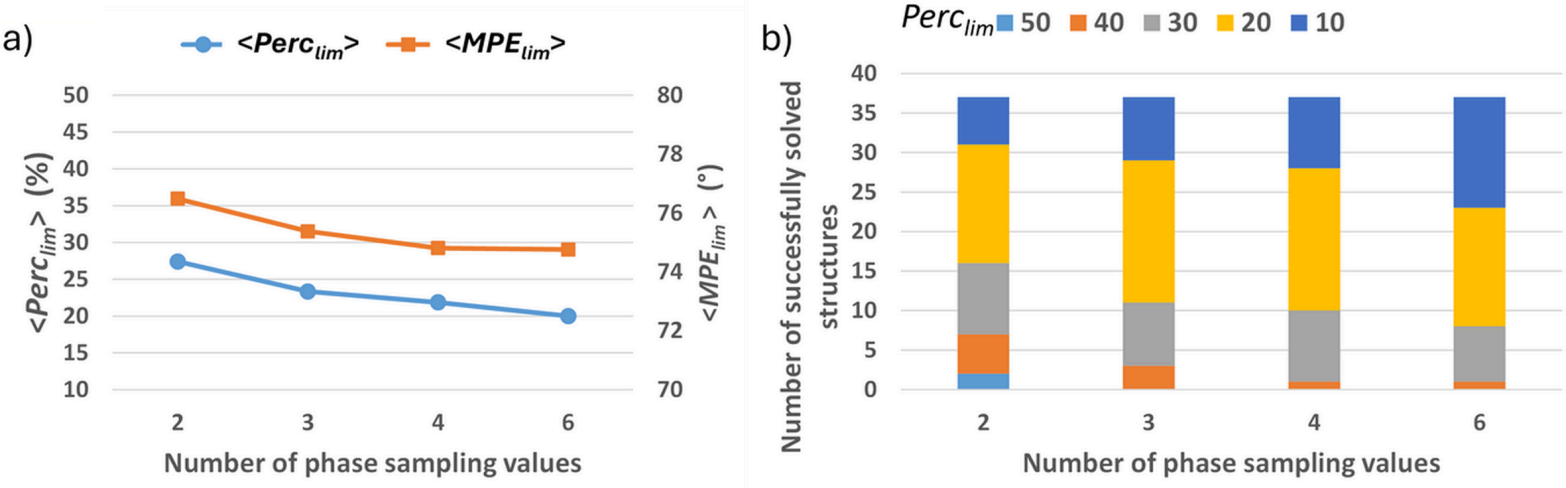
Results of the phase-seeding procedure on the full set of large test structures used in this study. (*a*) Perc_lim_ (left axis) and MPE_lim_ (right axis) and (*b*) the number of successfully solved test structures plotted for Perc_lim_ values (%) ranging from 10 to 50 as a function of the sampling density.

By averaging across all the large structures, higher values of Perc_lim_ and lower values of MPE_lim_ are obtained compared with medium structures [Fig. 9 ▸(*a*)]. The fraction of structures solved by using Perc_seed_ = 30 increases with respect to the case of medium structures, the distribution of Perc_lim_ is nearly similar when using 3 and 4 phase values, and the best results are obtained when using 6 phase values [Fig. 9 ▸(*b*)].

The dependence of the MPE resulting from the pre-processing step is in line with what was observed for small and medium structures (Figs. S8, S9). However, the results of the phasing step reveal some differences: the Perc_lim_ values are between 10% and 40% and have a positive correlation with data resolution (Res) [Fig. S10(*a*)], but surprisingly they have a positive correlation also with *Z*_max_ [Fig. S10(*b*)], in contrast to what was observed for small and medium structures. The corresponding MPE_lim_ values, between 60° and 85°, are consistent with a similar trend shown as a function of Res and *Z*_max_ [Figs. S10(*c*), S10(*d*)]. This anomaly can be explained by the role of heavy atoms in proteins, which are specifically introduced to enhance the phasing process, a necessity for crystals that diffract at low resolution. As a result, the correlation coefficient between Res and *Z*_max_ for large structures is 0.74, significantly higher than that for medium (0.00) and small (0.20) structures, and the dependence of MPE_lim_ on *Z*_max_ reflects that on Res.

### 4.4. Global statistics

**Figure 10:**
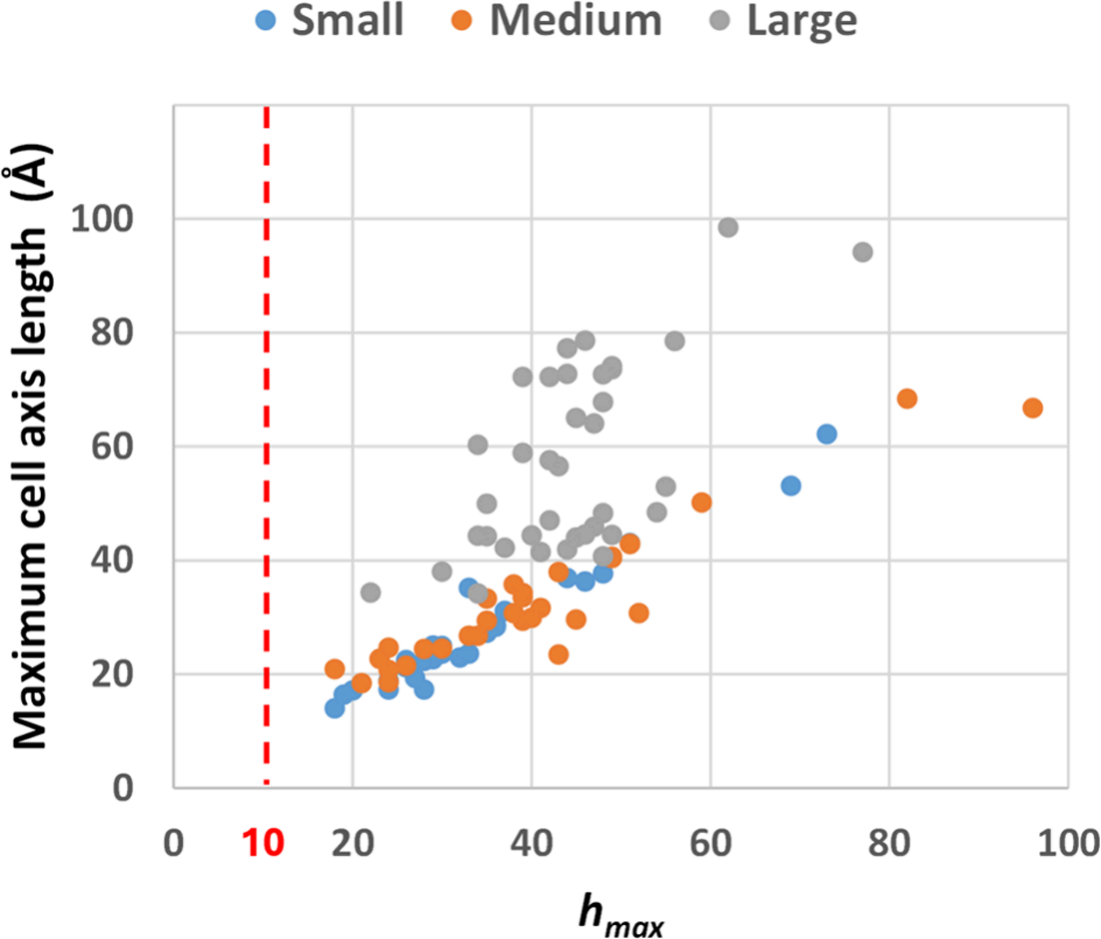
Maximum cell axis length versus the maximum Miller index for the small, medium and large test structures considered in this study. The value *h*_max_ = 10 is shown by the dashed line.

A comparison across small, medium and large structures reveals that none of the test structures considered in this study could be solved by the neural network developed by Larsen *et al.* (2024 ▸). This limitation arises not only due to the non-centrosymmetric nature of our test structures, but also because the network was trained with input data limited to a maximum Miller index value of 10. As shown in Fig. 10 ▸, the minimum value of the maximum Miller index is 22 for large structures, and 18 for medium and small structures, well above the limit of 10 shown by the dashed red line.

**Figure 11:**
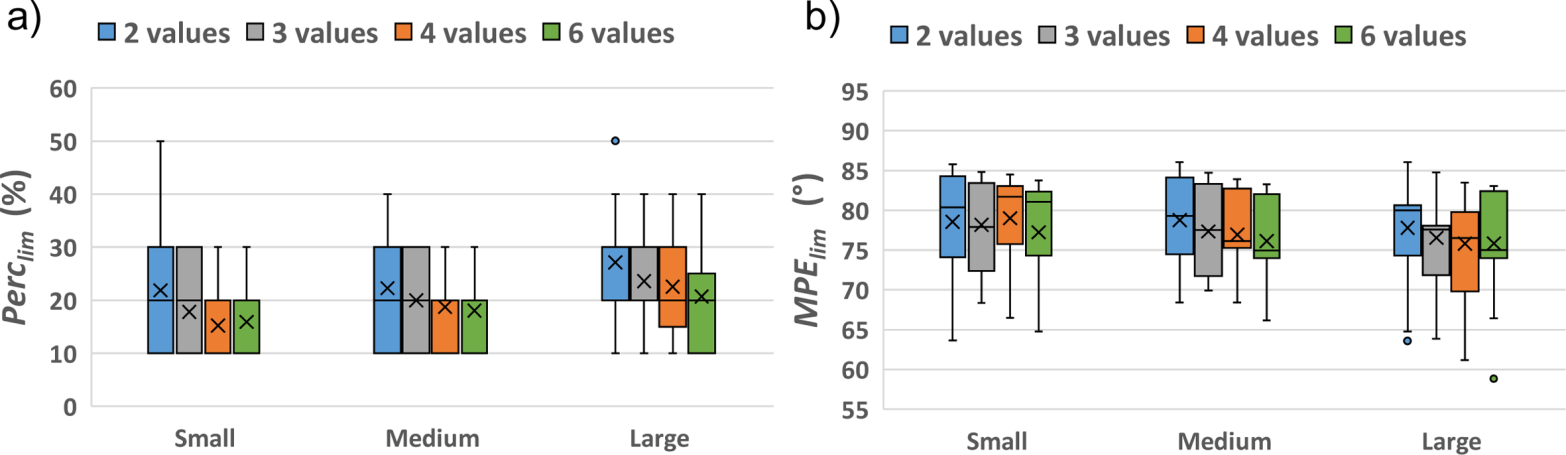
Box plot of the seed size (*a*) and of the MPE (*b*) that lead to a structure solution, calculated for the set of small, medium and large test structures for sampling densities from 2 to 6. The mean values of the distributions are shown by crosses.

The results of the phase-seeding procedure are summarized in Fig. 11 ▸, where a slight increase in the mean Perc_lim_ values can be seen when moving from small to medium and then to large structures, as shown in Fig. 11 ▸(*a*). Concerning the dependence on the sampling density, it can be deduced that the use of 2 values is not recommended due to the systematically higher Perc_lim_ values. Smaller values can be obtained when using 3 values, but the best option is between 4 and 6 values. For small and medium structures the performance of 4 and 6 values is very similar, so 4 is a preferred sampling density for computational resource arguments, while for large structures the 6 values lead to better results. The mean MPE_lim_ values in Fig. 11 ▸(*b*) follow an opposite trend, showing a slight decrease moving from small to large structures and from lower to higher sampling densities. The use of 6 values produces the lowest MPE_lim_ values, but the dependence on the sampling density is not as clear as for Perc_lim_. Actually the dependence of MPE_lim_ on sampling density is affected by two contributions: one arising from the size of the phase seed, the other due to the discretization of the continuous angular variable. Therefore, MPE_lim_ cannot serve as a reliable criterion for determining the best sampling density.

### 4.5. Powder data

**Figure 12:**
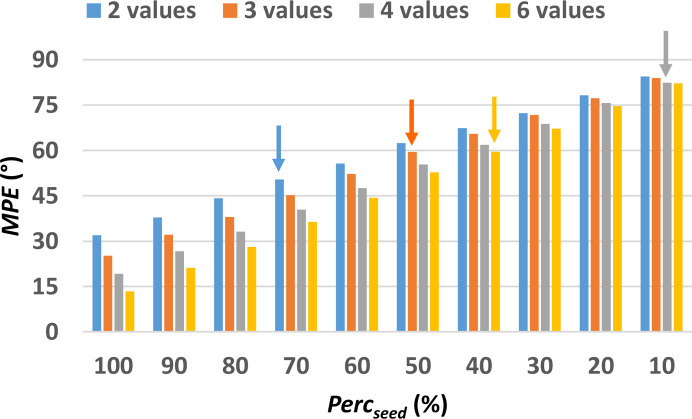
MPE of the discretized phase values with respect to the true values as a function of the size of the phase seed, measured by Perc_seed_, *i.e.* the percentage of reflections to which the true discretized phase value has been assigned. The lowest Perc_seed_ for which the initial set of phases converged towards a solution are highlighted by arrows. Calculations have been performed on the test structure with code name Bamo.

Applying phasing methods to powder diffraction data presents additional challenges compared with the single-crystal analysis. One key difficulty is the extraction of reflection intensities from the experimental X-ray diffraction pattern, which is complicated by intrinsic characteristics of the powder profile, especially the unavoidable overlap of diffraction peaks. Table S5 reports the *Rf* value calculated between extracted and true structure-factor amplitudes for the powder data structures considered in this study. The average value is 43%, to be compared with the values of 9%, 11% and 20% obtained for *Rf* calculated between measured and true structure factors for, respectively, small, medium and large single-crystal structures. As concerns seed phasing, this degradation of the input information does not affect the MPE values and their dependence on Perc_seed_ (Fig. 12 ▸), which are similar to those seen for small single-crystal structures (Fig. 4 ▸); rather, it greatly influences the phasing process. In fact, the output of the pre-processing step, shown in Fig. S11, is comparable with that observed for small single-crystal structures. Nevertheless, only two structures out of the four listed in Table S4 are solved by phase seeding: Bamo and ampicillin. They have the highest data resolution and contain heavy atoms (Ba and Mo for Bamo, S for ampicillin) which act as a pivot for the phasing process. Even for these structures, the solution is reached for values of Perc_lim_ (arrows in Fig. 12 ▸ for Bamo) that are much higher than those encountered for small single-crystal structures. Adopting different seed generation modes does not improve the phasing process. Bamo and ampicillin can be solved *ab initio* by DM implemented in *EXPO2013*.

For the other two structures, Aldx and Theoph, a partially correct structural fragment was obtained by using seed size with Perc_lim_ > 50%. These structural fragments are similar to those obtained by DM in *EXPO*. However, attempts to complete these partial structures through Fourier-recycling procedures, as implemented in the *EXPO2013* program (Altomare *et al.*, 2008 ▸, 2012 ▸), were unsuccessful. Even with Perc_seed_ values set to 100%, the complete fragment could not be recovered. This indicates that the use of discrete values for the phases of non-centrosymmetric structures is unable to improve the poor information on structure-factor vectors contained in the extracted intensities. The significant error on the extracted structure-factor magnitudes hinders the effective propagation of the phase seed.

### 4.6. Alternative ways to generate the phase seed

In the results presented so far, the reflections used to form the seed have been chosen randomly among the measured symmetry-independent ones. In this section we explore different ways of selecting reflections for the seed, based on reflection variables relevant to the phasing process. They are the resolution (*d*), given by
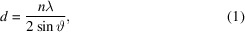
where λ is the primary X-ray beam wavelength and 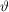 is the secondary X-ray beam scattering angle (Giacovazzo, 2014 ▸), and the normalized structure factor, defined by
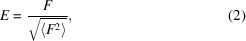
where *F* is the modulus of the structure factor of a given reflection, and the average at the denominator is calculated over all the reflections (Giacovazzo, 2014 ▸). *E* values have the advantage of being independent on the resolution of the reflection and have the property that
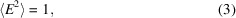
(Giacovazzo, 2014 ▸). Therefore, reflections can be identified by three key variables: the Miller indices (*hkl*), which determine their position within the reciprocal-lattice grid; the resolution value (*d*), which depends on the radial distance of the reflection from the centre of the reciprocal lattice; and the *E* value, which corresponds to the reflection intensity normalized to the scattering power of the specific crystal. These variables are weakly correlated, as can be seen in Fig. S12, and can therefore lead to selections of reflections very different from each other. We implemented the following alternative criteria for seed generation:

(i) *Random*: the criterion already adopted, where reflections are chosen randomly among the *N*_refl_ measured ones. The number of reflections is determined by fixing the percentage with respect to *N*_refl_.

(ii) *hkl-Sorted*: the size of the seed is fixed by defining the limit of the reciprocal lattice that contains it. All reflections are contained in the cubic grid having maximum Miller index *h*_max_ = 5, 10, 15, 20 or 25.

(iii) *d-Sorted*: reflections are sorted according to their data resolution, then the seed is formed by taking all reflections from the lower to higher resolution. The number of reflections is determined by fixing the percentage with respect to *N*_refl_.

(iv) *E-Sorted*: reflections are sorted according to their *E* values, then the seed is formed by taking all reflections from the higher to the lower *E* value. The number of reflections is determined by fixing the percentage with respect to *N*_refl_.

(v) *E-Random*: the reflections are chosen randomly among those having large *E* values (*E* > 1.0). The number of reflections is determined by fixing the percentage with respect to *N*_refl_.

**Figure 13:**
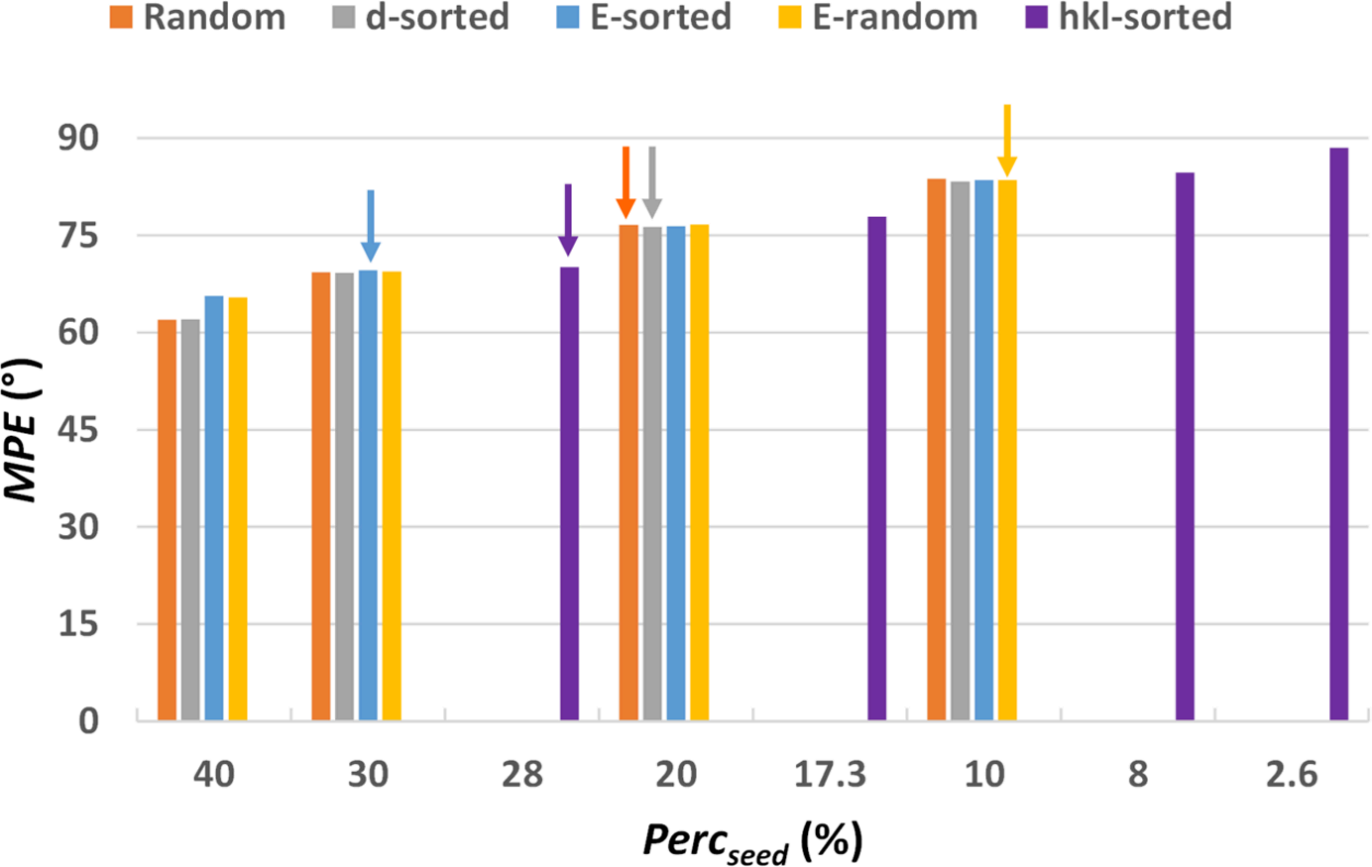
MPE of the discretized phase values with respect to the true values as a function of the size of the phase seed, measured by Perc_seed_, calculated for different ways of generating the phase seed. The Perc_seed_ values of 2.6, 8.0, 17.3 and 28.0 for the *hkl*-sorted seed generation are not in scale on the *X* axis and correspond to *h*_max_ = 10, 20, 25 and 30, respectively. The lowest Perc_seed_ for which the initial set of phases converged towards a solution are highlighted by arrows. Calculations have been performed on the large test structure with PDB code 193l, the first of those listed in Table S3.

The efficacy of these criteria to generate the phase seed is compared in Fig. 13 ▸, using a single-crystal structure (the protein with PDB code 193l). It can be noted that the MPE values mainly depend on the seed size (Perc_seed_), while they are only slightly affected by the selection criterion used for seed generation. However, the selection criterion significantly affects the efficiency of the phasing procedure. In fact, the *hkl-sorted* strategy results in a successful structure solution only when using a maximum value for the Miller indices *h*_max_ = 25, which corresponds to Perc_seed_ = 28%. Similarly, the *E-sorted* strategy does not appear particularly efficient, as the structure solution is reached only by using a large seed (Perc_seed_ = 30%). The *Random* and *d-sorted* criteria show equivalent effectiveness, both leading to a successful structure solution at Perc_seed_ = 20%. However, the highest efficiency is obtained by the *E-random* strategy, for which the structure solution is reached by using a small seed (Perc_seed_ = 10%).

**Figure 14:**
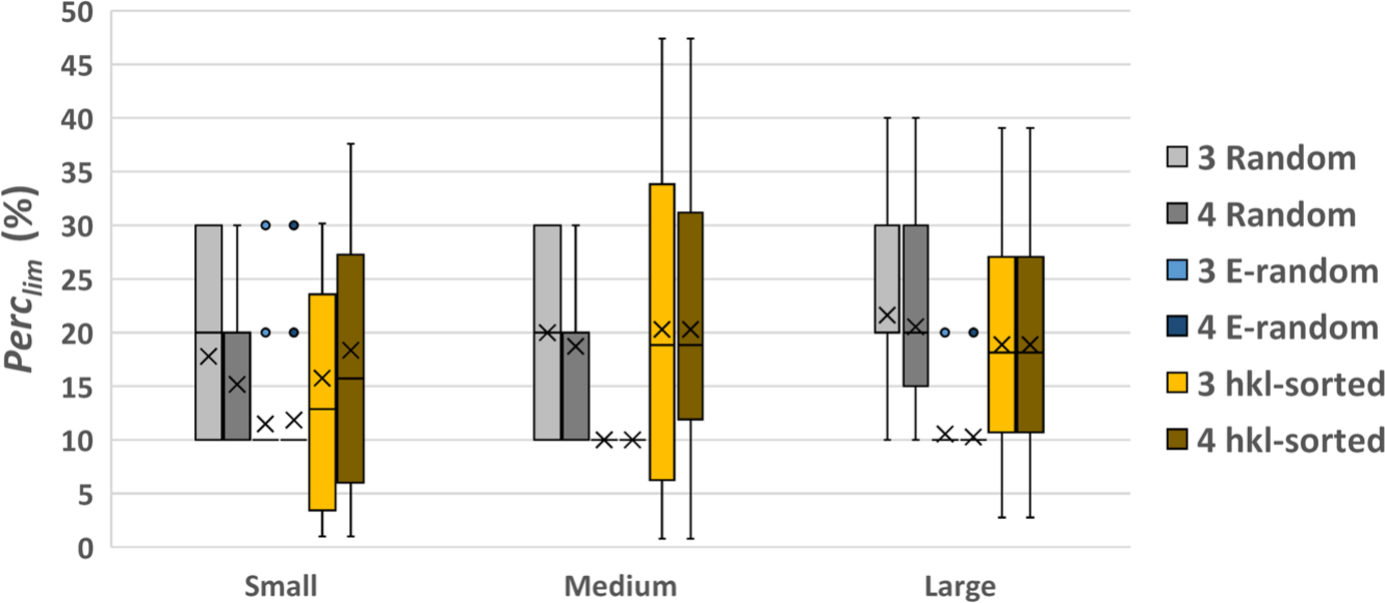
Box plot of the seed size (Perc_lim_) that leads to a successful structure solution, calculated for the set of small, medium and large test structures for sampling densities 3 and 4. The mean values of the distributions are shown by crosses.

To verify the generality of these results, we compare in Fig. 14 ▸ the Perc_lim_ values obtained by applying the above selection criteria for seed generation to all the test structures. In particular, we compare the *Random* approach, which has been our default method, with the *E-random* one, which was the best performing for the protein with PDB code 193l, and the *hkl-sorted* one, which ensures a localization of the seed within the reciprocal space. Fig. 14 ▸ shows that *E-random* remains the most effective strategy when all test structures are considered, particularly for large and medium structures. The *Random* and *hkl-sorted* strategies appear less efficient, as they require a larger number of reflections to reach a successful structure solution. It is interesting to observe the trend followed by the Perc_lim_ values when going from small to large structures. For the *Random* seed generation, they increase, indicating that the algorithm loses efficiency when processing larger structures. A similar, though less defined, trend is observed for the *hkl-sorted* seed generation. It should be noted that for this specific seed generation mode the Perc_lim_ value cannot be fixed, but it depends on the threshold applied in reciprocal space (*h*_max_). The corresponding averaged *h*_max_ values obtained for small, medium and large structures are 8.9, 10.8 and 15.3, respectively. Instead, for the *E-random* seed generation the efficiency of the phase-seeding protocol remains substantially constant across structure sizes. Another interesting result is the dependence on the sampling density among the seed generation criteria. In this study, we tested only intermediate sampling densities with 3 and 4 points, which showed negligible differences when using the *E-random* and *hkl-sorted* criteria. Notably, for these two latter seed generation modes, the 3-points sampling is more effective than the 4-points sampling for small structures.

**Figure 15:**
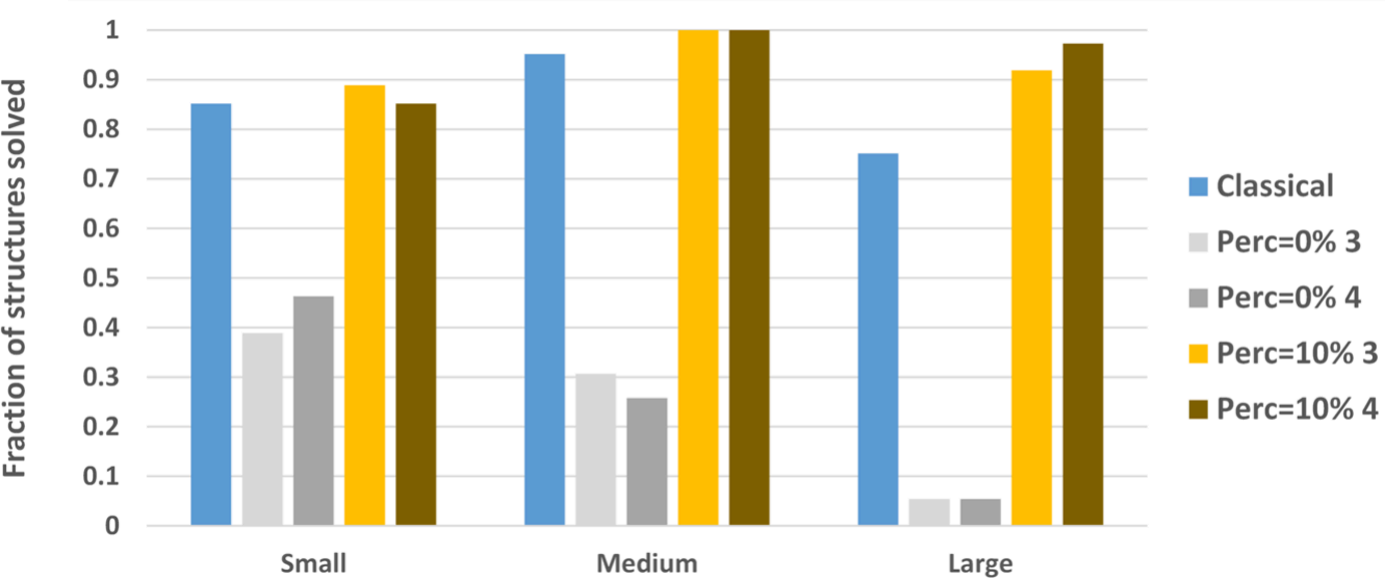
Fraction of successfully solved small, medium and large test structures by applying standard phasing procedures (classical), all-random initial phases (Perc_seed_ = 0%) and phase seeding with Perc_seed_ = 10%. Only sampling densities 3 and 4 are considered.

The overall efficiency of the phase-seeding procedure in solving the full set of test structures is assessed by considering the best-performing criterion for seed generation, which was found to be *E-random*. Since in this case most of the test structures are solved using Perc_seed_ = 10% (the Perc_lim_ distribution is significantly reduced around the value of 10% in Fig. 14 ▸), we used this size of the phase seed as a benchmark to assess the efficiency of the phase-seeding procedure. The results, shown in Fig. 15 ▸, are compared with those obtained using the classical *ab initio* phasing procedures (classical), which correspond to DM for small and medium structures, and Patterson methods for large structures, and using Perc_seed_ = 0%, *i.e.* by initiating the EDM cycles with random phases assigned to all reflections. It can be noted that the random phase assignment exhibits low efficiency, ranging from ∼40% for small structures to ∼5% for large structures and, as expected, it is outperformed by the phase assignment through direct or Patterson methods. Phase seeding with a seed size limited to Perc_seed_ = 10% successfully solves all medium and nearly all large test cases, demonstrating a higher efficiency than classical phase methods for these structures. The complete set of test structures can be successfully solved by phase seeding by increasing the number of seed reflections up to Perc_seed_ = 30%.

## 5. Discussion

Following the proof-of-principle application of AI to phase crystal structures by Larsen *et al.* (2024 ▸), we searched for ways to overcome the limitations of this new approach, which included the small size of crystal structures that were studied (with maximum unit-cell axis length <10 Å)^1^, the large number of structures required for AI training and the application to only non-centrosymmetric space groups. Our idea is to use the AI approach to pre-process diffraction data, with the aim of generating a seed of reflections whose phase values are closer to the true ones. This pre-processing step is based on two pillars: (i) discretization of phase values and (ii) generation of a seed of reflections with reliable (discretized) phase values. Following the pre-processing step, the task of extending this information to the full set of reflections and further refining the phase values is accomplished by phasing programs developed so far without the use of AI, based on recycling procedures in direct and reciprocal spaces. The results of our feasibility study indicate that this approach can actually lead to the successful solution of even large-scale non-centrosymmetric crystal structures, provided that the phase values are sampled by using only 3 or 4 points in the interval [0, 2π] and that the number of seed reflections is between 10% and 30% of *N*_refl_. Indeed, the observation that MPE of the seed does not depend on the characteristics of the structure and that Perc_lim_ is even lower for large structures implies that the phase-seeding procedure could be more efficient for large structures. Notably, we have also demonstrated that with a proper calibration of the seeding parameters we can make the demand on AI performance less challenging.

The results obtained by using a random choice of seed reflections (*Random* seed generation mode) have a double interpretation. For example, the fact that the structure solution is found with Perc_seed_ = 10% can be interpreted considering (i) a 100% efficient AI procedure applied to phase a seed formed by 10% of the *N*_refl_ reflections, or (ii) a 50% efficient AI procedure applied to phase a seed formed by 20% of the *N*_refl_ reflections. Consequently, the seed phasing protocol can be optimized by balancing the accuracy of the AI with the size of the phase seed, namely the number of reflections to be phased by AI. The first choice requires more elaborated AI strategies and larger training sets, while the second one demands more computational resources.

The *hkl-sorted* seed generation mode has been used to follow the same approach adopted by Larsen *et al.* (2024 ▸). This mode represents the best solution for technically applying AI, as it fixes the grid to be used to generate the diffraction patterns of the training and inference structures. However, we have seen that it is not the most effective solution for implementing the phase seeding (Fig. 14 ▸), and it does not allow for an extension towards increasingly larger structures (in fact, the test structure with PDB code 193l, which has a large unit cell, is solved only by using *h*_max_ = 25).

Seed generation strategies based on collecting reflections in the order in which they are sorted according to relevant variables for phasing, such as resolution (*d*) or normalized intensity (*E*), overcome the limit of the *hkl*-*random* approach. However, they seem to be less efficient than the strategies based on a random choice of the reflections (Fig. 13 ▸). The best strategy for generating the phase seed combines random selections of reflections with a selection based on their normalized intensity. In fact, the *E-random* approach, where seed reflections are chosen randomly from those with *E* > 1.0, was our initial attempt to select seed reflections. This aligns with a well known saying of Professor Giacovazzo: ‘the first foolish thing you do is always the best’. On the other hand, it is well known that normalizing the structure factors enhances the efficiency of phasing methods and standardizes their application to structures with varying atomic composition (Giacovazzo, 2014 ▸).

The practical use of the *E-random* approach for seed generation, as well as any approach not based on a fixed grid, poses challenges in applying AI, as it requires the development of specific protocols to standardize the AI input data derived from the measured and calculated diffraction patterns. However, in our view, this represents the best solution to guarantee the application of AI to the solution of increasingly larger structures.

Regarding data resolution, Fig. S1 illustrates that crystal structures can be realistically solved by using AI alone, trained with a cubic reciprocal lattice of size 10, only if low-resolution data are considered, *i.e.* Res lower than 4–6 Å for small–medium structures and 8–10 Å for large structures. However, high-resolution data are essential to correctly interpret electron-density maps in terms of chemical bonds. For this reason, in this study, we selected test structures biased towards the highest experimentally achievable resolution. In practice, these structures were chosen from those originally used to develop classical phasing methods. This choice is taken to demonstrate that our method provides a promising alternative to the structure-solving approaches currently in use. While these existing methods are effective in many cases, they often struggle with large-scale structures, low-resolution or incomplete data. By carefully selecting the appropriate seed reflections and their percentage, our method can be tested on classes of structures that are difficult to address with current approaches.

Applying phase seeding to powder data is challenging because the uncertainties inherent in structure-factor magnitudes, extracted from powder profiles, combine destructively with the approximations introduced when discretizing phase values for non-centrosymmetric structures. As a result, the structure solution cannot be achieved even when considering discretized phase values assigned to all the extracted reflections (Perc_seed_ = 100%). This finding aligns with the common difficulties encountered in solving powder structures using traditional phase methods, particularly when the degree of peak overlap is significant and the experimental resolution is far from atomic.

Finally, we note that, in this study, we applied phase seeding in a straightforward way, by treating the pre-processing and phasing steps as independent. In other words, we applied the standard phasing procedure followed by *ab initio* phasing as implemented in the computer programs *SIR2014* and *EXPO2013*, for single-crystal and powder data, respectively. A significant development of the phase-seeding strategy would put in synergy the two steps, for example, by increasing the weight of the seed reflections in the EDM procedure.

## 6. Related literature

The following references are cited in the supporting information: Clegg & Teat (2000 ▸), Burley *et al.* (2006 ▸), Werner *et al.* (1996 ▸), Fischer *et al.* (2016 ▸).

## 7. Conclusions

Artificial intelligence is becoming increasingly pervasive in crystallography, assisting in solving a wide range of problems connected to the experimental and computational aspects of structural investigations. However, until now, the main problem in crystallography, *i.e.* the phase problem, had not been directly addressed. Larsen *et al.* (2024 ▸) developed a proof-of-principle study that demonstrated that AI can associate phase values to a set of measured intensities. This has the potential to revolutionize *ab initio* crystal structure determination, but the study has only been applied to centrosymmetric structures containing a few atoms in the asymmetric unit, a class of structures that is currently routinely solved by DM. Extending the AI approach to larger structures, and to non-centrosymmetric crystal symmetries, for which the full angular range [0, 2π] must be explored for most phase values (except for restricted phase values, when present), is expected to require significant computational resources.

In this study, we propose a method to overcome the limitations of a purely AI-driven approach, by combining AI with standard *ab initio* procedures for crystal structure solution, based on probabilistic or Patterson-based methods, through a protocol called phase seeding. We demonstrate that a phase seed is sufficient to phase the entire structure, *i.e.* it is possible to reach the correct structure by assigning discrete phase values close to the true ones to a minimal subset of reflections. In this context, AI is used to pre-process crystallographic measurements and provide a seed of reflections with reliable phase values to phasing programs. Investigations into the optimal size and nature of the seed have revealed that sampling the [0, 2π] interval with only 3 points and assigning good phase values to 10% of the symmetry-independent reflections yields a structural solution with almost the same efficiency for small, medium and large structures.

The major novelties of this study lie in the possibility to solve the phase problem of non-centrosymmetric structures of any size through the process of phase discretization, and to select the reflections to be considered as seeds based on their normalized structure-factor amplitudes. Moreover, the study establishes limits on the accuracy and extent of AI calculations necessary to pre-process crystallographic data, by defining the optimal number of reflections required for phase assignment in relation to the total number of symmetry-independent reflections.

Seed phasing has the potential to extend the AI approach to solving *ab initio* crystal structures of any complexity and symmetry by taking advantage of the latest findings in the development of classification algorithms while also profiting from the availability of mature and robust classical phasing procedures.

We believe that the neural architecture most compatible with the phase-seeding method will differ from the one developed by Larsen *et al.* Their approach is tailored for centrosymmetric structures and has been trained on a complete set of reflections with Miller indices with maximum *h*, *k*, *l* values restricted to 10, 10, 10. In contrast, the AI framework suitable for our method could be effectively designed and trained on a subset of reflections. This subset could be complete or incomplete, depending on whether *hkl-sorted* or *E-random* seed generation is employed, and the maximum Miller indices would not necessarily be constrained to (10, 10, 10).

Future work will focus on testing Larsen *et al.*’s neural network in combination with the phase-seeding method as well as developing a new AI framework capable of handling both centrosymmetric and non-centrosymmetric structures.

## Supplementary Material

Supporting information. DOI: 10.1107/S2053273325002797/lu5043sup1.pdf

## Footnotes

1 Given the relationship between crystal dimensions in direct and reciprocal space (see Section S2), the size of the crystal cell is directly proportional to the number of lattice nodes that are considered for phasing, thus to the complexity of the input for AI.
