# Supplementary material for: The phase-seeding method for solving non-centrosymmetric crystal structures: a challenge for artificial intelligence
Source: Acta Crystallogr A Found Adv. 2025 Apr 17;81(Pt 3):188–201. doi: 10.1107/S2053273325002797 (PMC12053495; doi:10.1107/S2053273325002797)
Supplement: Supplementary file 1 [file a-81-00188-sup1.pdf]

FOUNDATIONS
ADVANCES

Volume 81 (2025)

Supporting information for article:

The phase-seeding method for solving non-centrosymmetric crystal structures: a challenge for artificial intelligence

Benedetta Carrozzini, Liberato De Caro, Cinzia Giannini, Angela Altomare and Rocco Caliandro

S1. Properties of test structures

Table S1 Crystallographic information of small test structures.

COD id	N_{asym}	Cell content	Space group	Data resolution (Å)	Crystal cell axis length (Å)	Maximum h,k,l values	N_{refl}
2225745	54	$H_{720}C_{828}N_{72}O_{36}Fe_{36}$	R 3 c	0.84	22.82 22.82 36.93	27 27 44	3247
2230280	22	H_{126} C_{78} N_6 O_{48}	P 6_1	0.83	13.26 13.26 16.46	16 16 19	1612
2103455	56	$H_{374}C_{205}N_{48}O_{83}$	P 6_1	0.78	28.72 28.72 9.90	36 36 12	5504
2226585	53	$H_{104}C_{120}I_8$	P -4 2_1 c	0.77	18.88 18.88 7.24	24 24 9	1690
2219630	15	$H_{20}C_{24}N_4O_2$	P n	0.77	5.75 6.24 14.02	7 8 18	1162
2104300	54	$H_{180}C_{264}N_{12}O_{48}$	P 3_2 2 1	0.84	11.87 11.87 43.05	14 14 51	3556
2222097	27.5	$H_{288}C_{316}N_{32}O_{76}Zn_{16}$	I -4 2 d	0.75	14.32 14.32 37.73	18 18 48	2583
2228788	32	$H_{448}C_{448}O_{32}S_{32}$	F d d 2	0.75	17.88 53.15 10.23	23 69 13	3062
2103727	66	$C_{234}Cl_{162}$	P 3 c 1	0.77	18.70 18.70 22.68	24 24 29	5269
2103750	14.6	$H_{96}C_{224}N_{32}O_{64}F_{192}Cu_{16}$	I 4_1 c d	0.77	18.59 18.59 22.35	24 24 28	2282
2016712	7	$H_{24}C_4N_8O_8Na_4Cl_4$	P n 2_1 m	0.61	6.44 5.17 17.30	10 8 28	1479
2016711	7	$H_{24}C_4N_8O_8Na_4Cl_4$	I 2	0.70	6.48 5.24 17.35	9 7 24	969
2230031	33.5	$H_{176}C_{192}N_{24}O_{44}Cu_8$	I 4	0.84	22.523 22.53 10.29	26 26 12	2434

2227526	38.4	$H_{246}C_{268}N_{16}O_{23}$	P -4 21 c	0.71	19.38 19.38 14.08	27 27 19	4230
2013502	9	$H_{120}C_{112}N_8O_{16}Fe_8$	F m m 2	0.77	9.95 36.30 7.06	12 46 8	816
2223241	14.1	$H_{400}C_{216}N_{18}O_2Cl_{18}$	R 3 c	0.77	28.3787 28.3787 8.5236	36 36 11	1514
2016619	67	$H_{124}B_8C_{180}N_{36}O_8F_{32}Co_4$	C c	0.77	13.30 12.13 27.30	17 15 35	4681
2217894	41.5	$H_{276}C_{288}N_{48}O_{138}K_{12}Tb_{12}$	P 6_5 2 2	0.84	12.72 12.72 62.23	15 15 73	3094
2229326	33	$H_{368}C_{240}O_{24}$	P 4_3 2_1 2	0.83	13.32 13.32 31.16	16 16 37	3012
2225554	24.5	$H_{504}C_{336}N_{72}O_{120}Na_6P_{24}S_{24}Nd_6$	P -4 3 n	0.71	22.94 22.94 22.94	32 32 32	3151
2016696	5	Mg76 Zn324 Ce80	F -4 3 m	0.80	21.20 21.20 21.20	26 26 26	544
2226240	50	H320 C336 O48 Sn16	I -4	0.77	23.60 23.60 13.85	30 30 17	4638
2219750	19.6	H480 C672 N192 O48 P8 S4 Ni16	I -4 3 d	0.81	24.96 24.96 24.96	30 30 30	1368
2101410	43.5	$H_{308}C_{224}N_{56}O_{56}S_8Cl_4$	C 2 2 21	0.93	10.62 17.04 35.21	11 18 33	1933
2218160	61.3	$H_{216}C_{324}N_{54}O_{126}Fe_{12}W_{36}$	R 3	0.84	25.089 25.09 17.23	29 29 20	3707
2218446	8	$H_{288}C_{128}O_{32}Tl_{32}$	P -4 3 n	0.84	17.15 17.15 17.15	20 20 20	829
2224920	67	$H_{320}C_{220}N_4O_{44}$	P n a 2_1	0.71	11.24 20.17 23.65	15 28 33	8021

Table S2 Crystallographic information of medium test structures.

COD/CCDC id	N_{asym}	Cell content	Space group	Data resolution (Å)	Crystal cell axis length (Å)	Maximum h,k,l values	N_{refl}
2012193	102	$H_{464}C_{264}N_{48}O_{96}$	$P\ 2_1\ 2_1\ 2_1$	0.77	12.3290<br>20.2450<br>31.6910	15 26 41	9869
2228715	259	$H_{892}C_{764}N_{48}O_{208}S_{16}$	$P\ 2_1\ 2_1\ 2_1$	0.59	22.810<br>26.470<br>30.770	34 44 52	47837
2222265	204	$H_{576}C_{528}N_{96}O_{192}$	C c	0.69	13.3461<br>66.7770<br>15.0195	19 96 21	21275
2104241	208	$H_{768}C_{320}N_{64}O_{416}Fe_{32}$	$B\ 2_1$	0.83	14.5070<br>14.2260<br>68.3900	17 17 82	25223
CCDC 152025	130	$H_{272}C_{196}N_{32}O_{32}$	$P\ 2_1$	0.82	13.4583<br>19.4036<br>21.5084	16 23 26	10902
2016443	121	$H_{432}C_{428}N_{16}O_{20}Cl_{16}Ru_4$	$P\ 2_1\ 2_1\ 2_1$	0.77	13.746<br>23.674<br>26.781	17 30 33	10717
2016444	121	$H_{432}C_{428}N_{16}O_{20}Cl_{16}Ru_4$	$P\ 2_1\ 2_1\ 2_1$	0.77	13.769<br>23.691	17 30 34	10851
CCDC 213578	116	$H_{160}C_{128}N_{16}O_{88}$	$P\ 2_1$	0.87	13.4266<br>17.8128<br>18.4482	15 20 21	6846
CCDC 213579	232	$H_{160}C_{128}N_{16}O_{88}$	$P\ 2_1$	0.93	13.4291<br>17.6940<br>35.7701	14 19 38	11368
2103880	219	$H_{448}C_{508}N_{16}O_{352}$	C 2	0.94	19.3190<br>24.1900<br>33.3150	20 25 35	9790

2200556	124	$H_{270}C_{162}N_{38}O_{40}S_8$	P 2_1	0.83	11.5563<br>9.9957<br>42.9208	13 12 51	8977
2226177	90	$H_{480}C_{264}B_{12}F_{48}P_{24}Rh_{12}$	P 2_1 2_1 2_1	0.84	10.2240<br>14.7710<br>50.1580	12 17 59	7360
2011222	141	$H_{252}C_{220}N_{12}O_{48}Ru_2$	P 2_1	0.76	18.510<br>15.083<br>18.577	24 19 24	12839
CCDC 223140	200	$H_{912}C_{568}N_{96}O_{136}$	P 2_1 2_1 2_1	0.82	10.871<br>39.468<br>40.693	13 48 49	17947
2200556	124	$H_{270}C_{162}N_{38}O_{40}S_8$	P 2_1	0.83	11.556<br>9.996<br>42.921	13 12 51	8977
2101407	89.5	$H_{486}C_{363}N_{60}O_{102}S_{12}$	P 6_5	1.01	22.196<br>22.196<br>24.640	22 22 24	3721
2202059	105	$H_{440}C_{224}O_{188}S_8$	P 2_1 2_1 2_1	0.75	14.464<br>17.337<br>29.388	19 23 39	9891
2226460	84	$H_{496}C_{416}N_{88}O_{144}S_{16}Gd_8$	A b a 2	0.84	33.5290<br>23.3375<br>15.2046	39 27 18	5462
2103676	109.5	$H_{550}C_{288}O_{150}$	P 2_1 2_1 2_1	0.65	10.936<br>25.530<br>29.640	16 39 45	16516
2103675	100.3	$H_{599}C_{252}O_{149}$	P 2_1 2_1 2_1	0.54	14.636<br>21.637<br>23.450	27 39 43	26008
2225069	120	$H_{480}C_{384}N_{32}O_{64}$	P n a 2_1	0.80	19.4356<br>11.0542<br>40.4107	24 13 49	8810

2202173	92	$H_{174}C_{162}N_2O_{20}$	P 2_1	0.85	10.404<br>34.308<br>9.516	12 39 11	5806
2012678	81	$H_{396}C_{220}N_{36}O_{68}$	P 2_1 2_1 2_1	0.85	14.139<br>18.739<br>24.473	16 22 28	6095
2012679	83	$H_{412}C_{228}N_{36}O_{68}$	P 2_1 2_1 2_1	0.81	14.245<br>18.870<br>24.558	16 22 30	6824
2230103	90	$H_{184}C_{128}N_8O_{40}Co_4$	P c	0.84	12.0201<br>20.7463<br>17.6353	14 24 20	5705
21022479	109	$H_{368}C_{224}N_8O_{204}$	P 2_1 2_1 2_1	0.81	15.069<br>17.650<br>29.439	18 21 35	8339
2010528	107	$H_{508}C_{284}N_{48}O_{96}$	P 2_1 2_1 2_1	0.80	13.2450<br>21.9840<br>30.7320	16 27 38	10035
2008512	110.8	$H_{500}C_{280}N_{52}O_{111}$	P 2_1 2_1 2_1	0.87	21.205<br>37.941<br>12.227	24 43 13	8571
2101409	94	$H_{360}C_{256}N_{48}O_{64}S_8$	P 2_1 2_1 2_1	1.14	20.940<br>18.530<br>18.800	18 16 16	2928
2101408	83.5	$H_{283}C_{219}N_{48}O_{51}S_8Cl_8$	P 2_1 2_1 2_1	0.95	16.054<br>17.128<br>22.706	16 18 23	4087
2100208	223.5	$H_{295}C_{283}O_{160}Cl_4$	P 2_1	0.74	10.9260<br>25.2840<br>29.9540	13 34 40	20382

Table S3 Crystallographic information of large test structures.

PDB id	N_{asym}	Heavy atom content	Space group	Data resolution (Å)	Crystal cell axis length (Å)	Maximum h,k,l values	N_{refl}
193L	994	Cl Na	P 4_3 2_1 2	1.33	78.540<br>78.540<br>37.770	56 56 23	24111
1A0M	242		I 4	1.09	44.400<br>44.400<br>23.500	40 40 21	9265
1AAC	806	Cu	P 2_1	1.30	28.950<br>56.540<br>27.550	22 43 21	21199
1AHO	501		P 2_1 2_1 2_1	0.96	45.940<br>40.680<br>29.930	47 42 29	31001
1ARM	2455	Cu Hg	P 2_1	1.13	51.700<br>60.320<br>47.200	27 34 26	26079
1B0Y	623	Fe_4	P 2_1 2_1 2_1	0.93	37.681<br>41.675<br>41.913	40 44 41	41951
1BX8	397		P 4_3 2_1 2	1.38	37.713<br>37.713<br>67.812	27 27 48	10468
1C75	496	Fe	P 2_1 2_1 2_1	0.97	37.144<br>39.422<br>44.021	38 40 45	38891
1CBN	323		P 2_1	0.83	40.763<br>18.492<br>22.333	48 17 26	28718
1FS3	940		P 3_2 2 1	1.35	64.046<br>64.046<br>63.355	47 47 46	33054

1GYO	2549	Fe_8	$P\ 3_1$	1.20	56.670<br>56.670<br>94.170	47 47 77	104634
1H87	998	$Cl_4\ Gd_2$	$P\ 4_3\ 2_1\ 2$	1.72	77.250<br>77.250<br>38.660	44 44 22	12871
1HHU	505.5	Cl_{16}	$P\ 2_1$	0.89	22.705<br>27.986<br>44.490	25 31 49	42290
1HHY	208.17	$Cl_{48}\ Na_{16}$	$P\ 6_3\ 2\ 2$	0.89	48.444<br>48.444<br>43.086	54 54 46	22768
1HHZ	353.5	Cl_{36}	$P\ 3_2\ 2\ 1$	0.99	48.290<br>48.290<br>39.300	48 48 39	29932
1ICK	249.8	$P_{40}\ Mg_3$	$P\ 2_1\ 2_1\ 2_1$	0.95	17.870<br>31.550<br>44.580	18 33 46	16102
1IGD	465		$P\ 2_1\ 2_1\ 2_1$		34.900<br>40.300<br>42.200	31 36 37	23498
1IKJ	1416	Fe	C 2	1.27	70.180<br>42.480<br>52.960	55 31 41	39432
1JES	450	$P_4\ Cu_2$	$P\ 2_1$	1.49	25.343<br>34.359<br>31.093	16 22 20	8520
1L0Z	1846	$Na\ Br_{32}\ Xe$	$P\ 2_1\ 2_1\ 2_1$		49.500<br>57.700<br>73.600	32 38 49	34428
1L1G	1822	$Na\ Br_8\ Xe$	$P\ 2_1\ 2_1\ 2_1$	1.50	50.280<br>57.700<br>74.110	33 37 49	34588

1M1F	1661	P	$P\ 2_1$	1.25	33.264 45.457 65.021	26 35 45	43111
1M77	225	Co	$P\ 4_3\ 2_1\ 2$	1.23	44.300 44.300 24.800	35 35 20	6143
1NKD	510		C 2	1.10	47.06 37.88 31.65	42 34 28	21972
1PAZ	930	Cu	$P\ 6_5$	1.55	50.000 50.000 98.500	31 31 62	19659
1PLC	732	Cu	$P\ 2_1\ 2_1\ 2_1$	1.33	29.600 46.860 57.600	20 34 42	14307
1U0X	1418	Fe Xe_2	C 2	1.45	70.196 42.461 52.990	48 29 35	26873
1W1D	1259	P_4 Au	$P\ 2_1$	1.50	35.399 58.922 36.578	23 39 24	23260
1YTT	1770	Yb_4	$P\ 2_1\ 2_1\ 2_1$	1.80	65.508 72.216 45.035	36 39 25	19677
2BF9	292	Zn	C 2	0.99	34.180 32.920 28.450	34 33 28	17058
2F14	2063	Zn Hg	$P\ 2_1$	1.71	42.260 41.600 72.220	24 24 42	25705
2FP1	2606	Pb_2	$P\ 2_1$	1.50	42.644 72.673 62.060	28 48 41	54526
2KNT	462	P	$P\ 2_1$	1.20	25.690 38.040 28.640	21 30 23	16000

2P09	1319	P_3 Cl Zn	P 3_2 2 1	1.65	72.791<br>72.791<br>54.752	44 44 32	20345
2YZW	2325	Gd_2	P 2_1 2_1 2_1	1.70	44.147<br>73.950<br>78.606	25 43 46	28809
362D	293	P_{40} Co_8	P 2_1 2_1 2_1	1.30	21.162<br>28.670<br>44.335	16 22 34	7026
3EBX	472		P 2_1 2_1 2_1	1.40	49.940<br>46.580<br>21.590	35 33 15	10445
8RXN	389	Fe	P 2_1	1.00	19.970<br>41.450<br>24.410	19 41 22	18529

Table S4 Crystallographic information of a few test structures solved from X-ray powder diffraction data.

Code name	N_{asym}	Chemical formula	Space group	Data resolution (Å)	Crystal cell axis length (Å)	Maximum h,k,l values	Ref.
Tetra	33	$C_{22}H_{25}N_2O_8^+ Cl^-$	*P* $2_1 2_1 2_1$	1.16	10.9300<br>12.7162<br>15.7085	9 11 13	Clegg & Teat, 2000
Ampicillin	27	$C_{16}H_{19}N_3O_4S{\cdot}3H_2O$	*P* $2_1 2_1 2_1$	0.99	15.52275<br>18.9256<br>6.67375	15 19 6	Burley *et al.,* 2006
Bamo	28	$BaMo_3O_{10}$	*P* 2_1	0.89	14.695<br>7.5704<br>6.9618<br>100.381	16 8 7	Werner *et al.*, 1996
Theoph	44	$C_7H_8N_4O_2{\cdot}$ C_7H_7NO	*P* 4_1	1.18	10.281404<br>10.281404<br>26.15892	8 8 21	Fischer *et al.*, 2016

W., Clegg and S. J., Teat, *Acta Cryst*. (2000), **C56**, 1343–1345,

J.C., Burley, J., van de Streek, P.W., Stephens, *Acta Cryst*, (2006), **E62**, o797–o799

P.-E. Werner, M. Moustiakimov, B.-O. Marinder, K. S. Knight, (1996), *Z. für Kristallographie*, **212**, 665-670

F., Fischer, M.U., Schmidt, S. Greisera, F.Emmerling, (2016), *Acta Cryst., Sect. C, Cryst. Struct. Commun*., **72**, 217-224

Table S5 For each powder test structure, the agreement factor between the extracted and true structure factor amplitudes (*Rf*) is calculated based on the number of symmetry-independent reflections (N_{refl}).

**Code name**	$Rf\,(\lvert F\rvert_{extracted}-\lvert F\rvert_{true})$	N_{refl}
Tetra	43.29	892
Ampicillin	45.71	1254
Bamo	43.28	1220
Theoph	37.82	906

S2. Unit cell dimensions

In case of cubic symmetry, the crystal cell parameter (a) is related to the maximum value of Miller index (h_{max}) and the data resolution (Res), by the following equation:

$$a = \frac{h_{max}\sqrt{3}}{Res} \qquad \text{(S1)}$$

Eq. (S1) is plotted in Figure S1 for three resolution values.

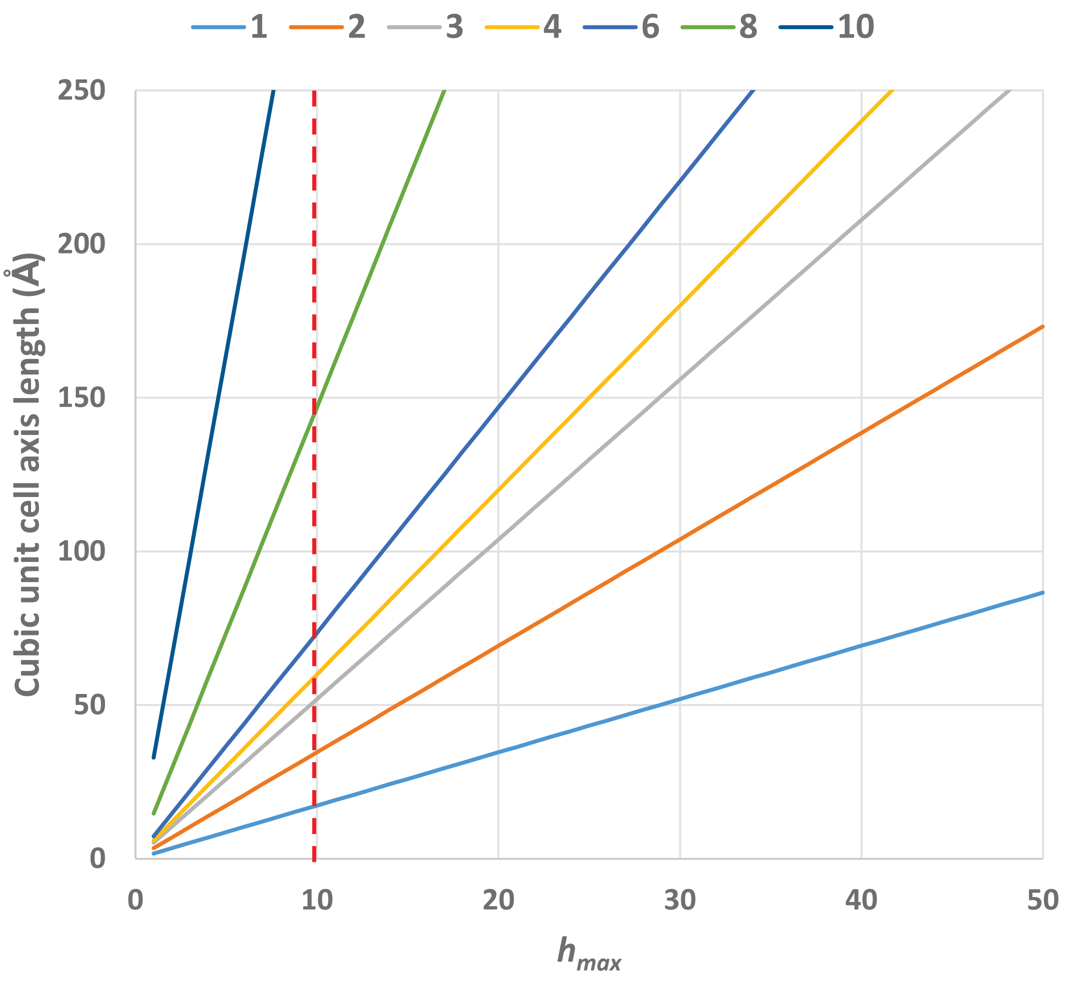

Figure S1 Scatter plot of maximum crystal cell length versus maximum Miller index in the hypothesis of cubic symmetry, calculated for data resolution values ranging from 1.0 Å to 10.0 Å.

S3. Tests on small structures

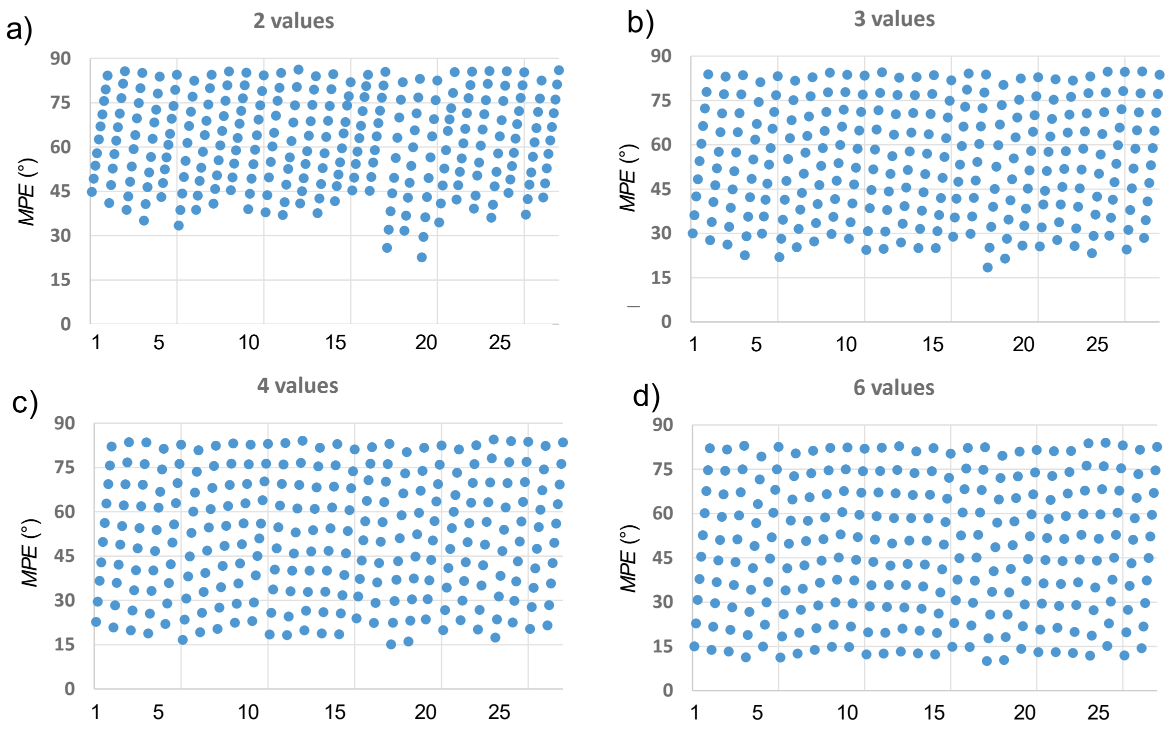

Figure S2 Mean phase errors obtained by using 2 (a), 3 (b), 4 (c) and 6 (d) sampling points for the phase values, calculated for all the small test structures used.

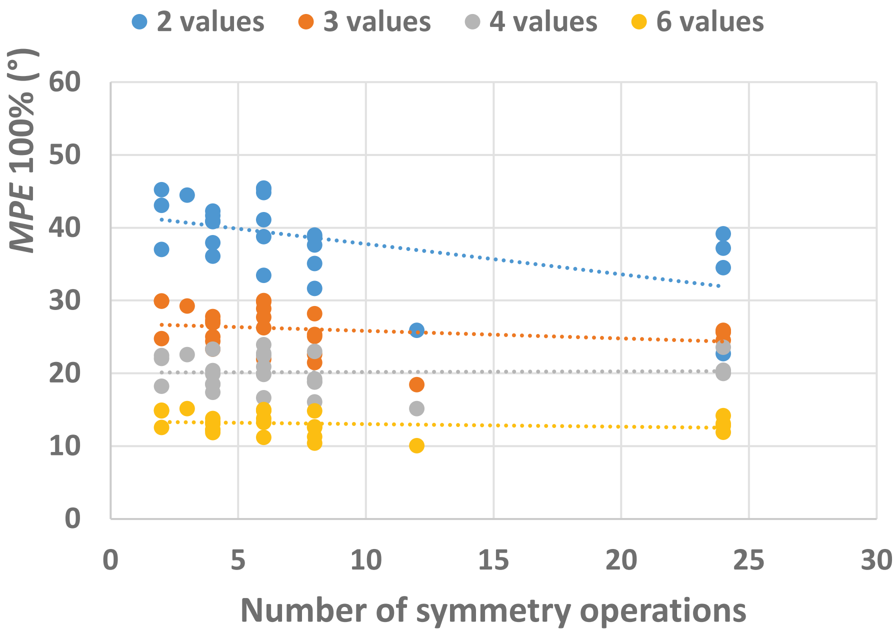

Figure S3 Mean phase errors of the maximum seed size, *i.e.* for $Perc_{seed}$=100% calculated for different sampling densities of the phase values as a function of the number of symmetry operations, calculated for all the small test structures.

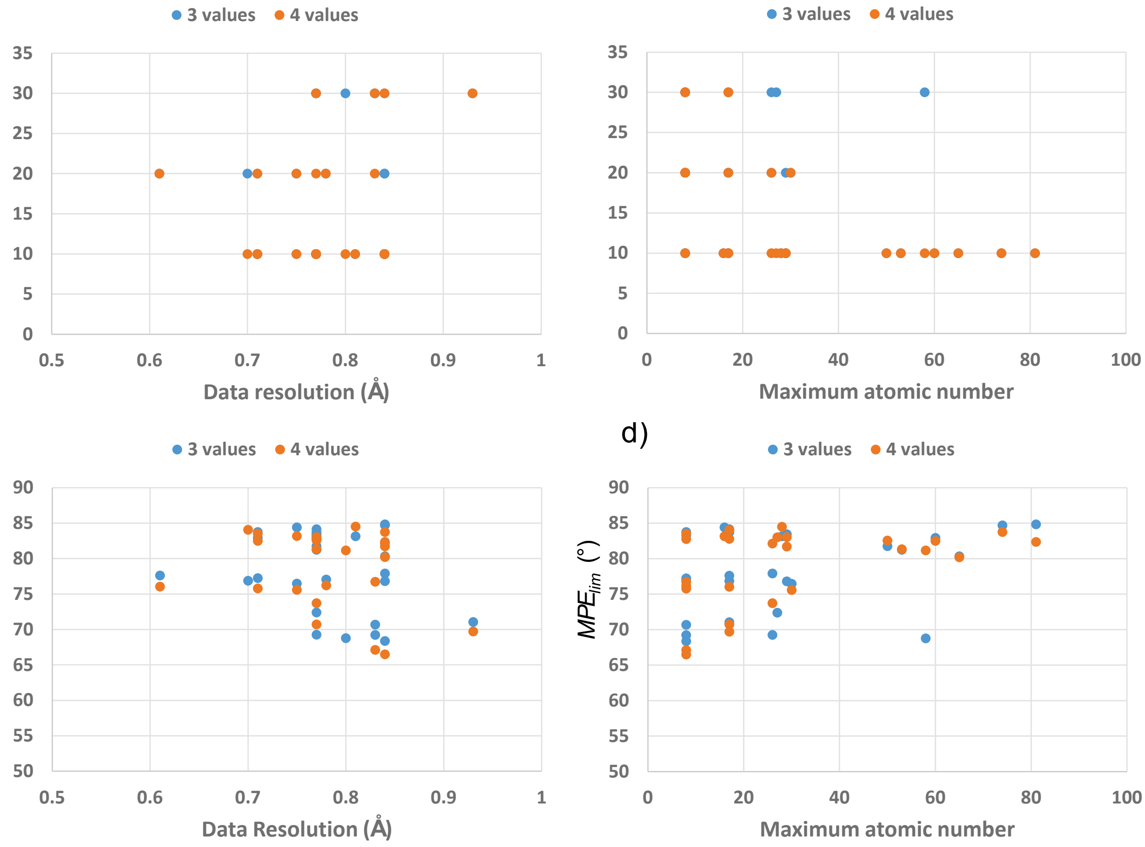

Figure S4 Minimum percentage of the number of seed reflections with respect to the total number of symmetry-independent reflections for which the phasing procedure leads to the correct structure solution ($Perc_{lim}$) (a, b) and maximum *MPE* for which the phasing procedure leads to the correct structure solution (MPE_{lim}) (c, d) obtained by using 3 (blue circles) and 4 (orange circles) sampling points for phase values, plotted as a function of the data resolution (a, c) and the atomic number of the heaviest atomic species present in the crystal (b, d), for all the small test structures used.

S4. Tests on medium structures

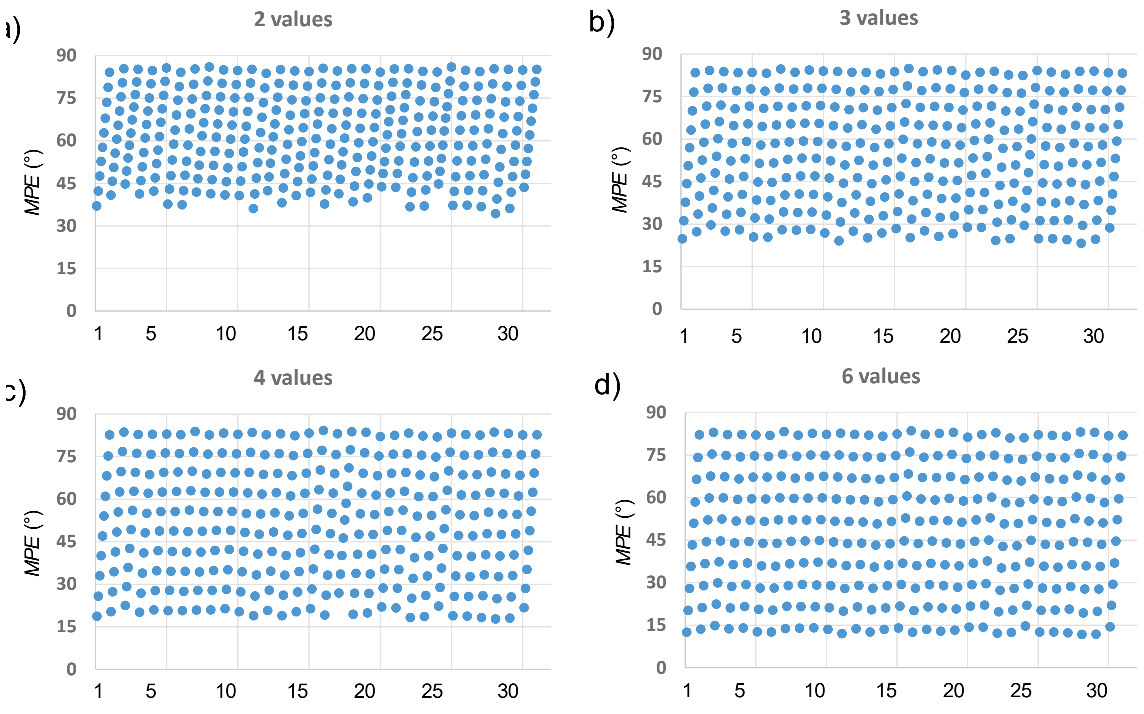

Figure S5 Mean phase errors obtained by using 2 (a), 3 (b), 4 (c) and 6 (d) sampling points for the phase values, calculated for all the medium test structures used.

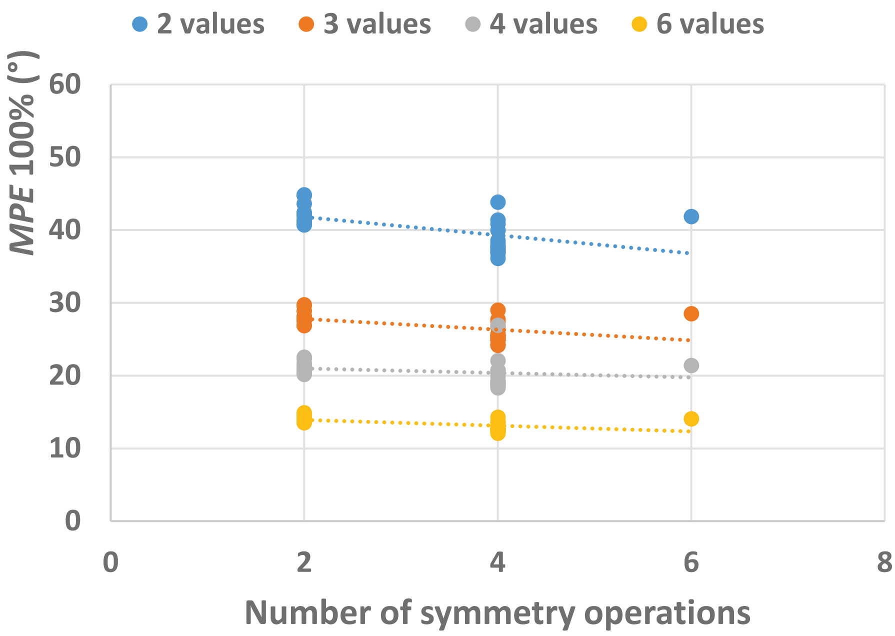

Figure S6 Mean phase errors of the maximum seed size, *i.e.* for $Perc_{seed}$=100% calculated for different sampling densities of the phase values as a function of the number of symmetry operations, calculated for all the medium test structures used.

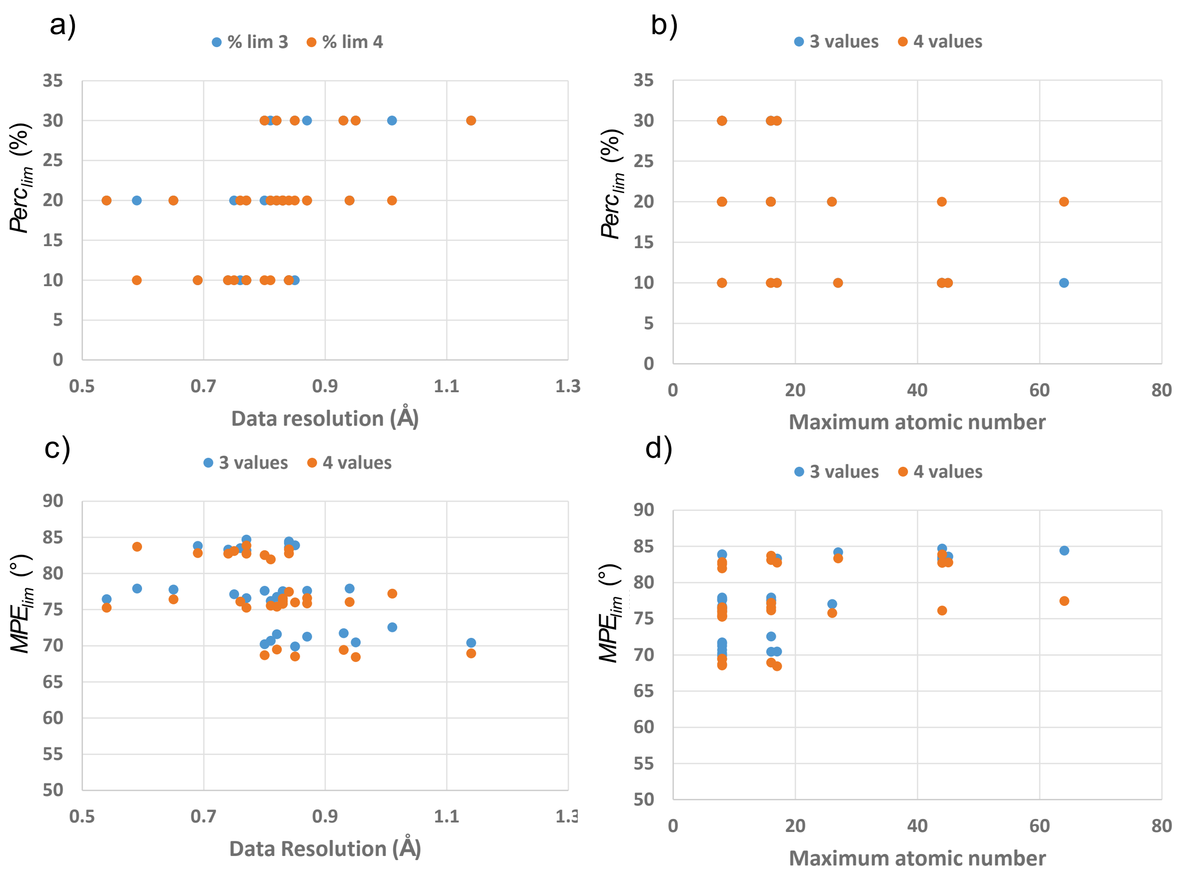

Figure S7 Minimum percentage of seed reflections with respect to the total number of symmetry-independent reflections for which the phasing procedure leads to the correct structure solution ($Perc_{lim}$) (a, b) and maximum *MPE* for which the phasing procedure leads to the correct structure solution (MPE_{lim}) (c, d) obtained by using 3 (blue circles) and 4 (orange circles) sampling points for phase values, plotted as a function of the data resolution (a, c) and the atomic number of the heaviest atomic species present in the crystal (b, d), for all the medium test structures used.

S5. Tests on large structures

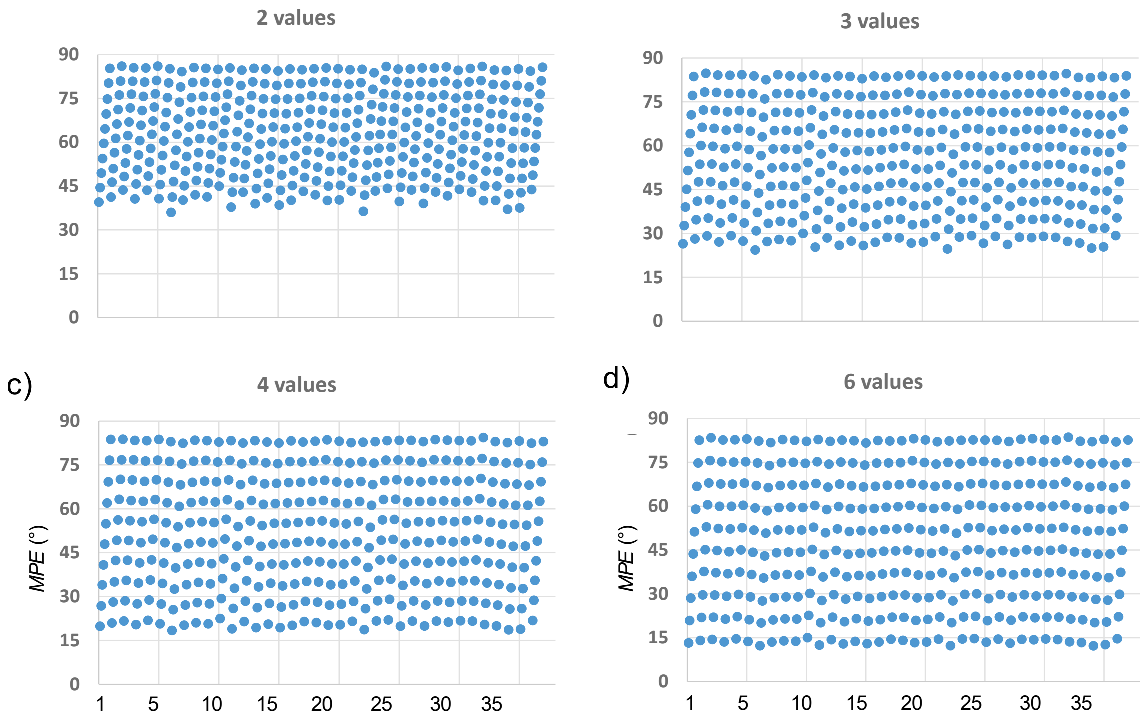

Figure S8 Mean phase errors obtained by using 2 (a), 3 (b), 4 (c) and 6 (d) sampling points for the phase values, calculated for all the large test structures used.

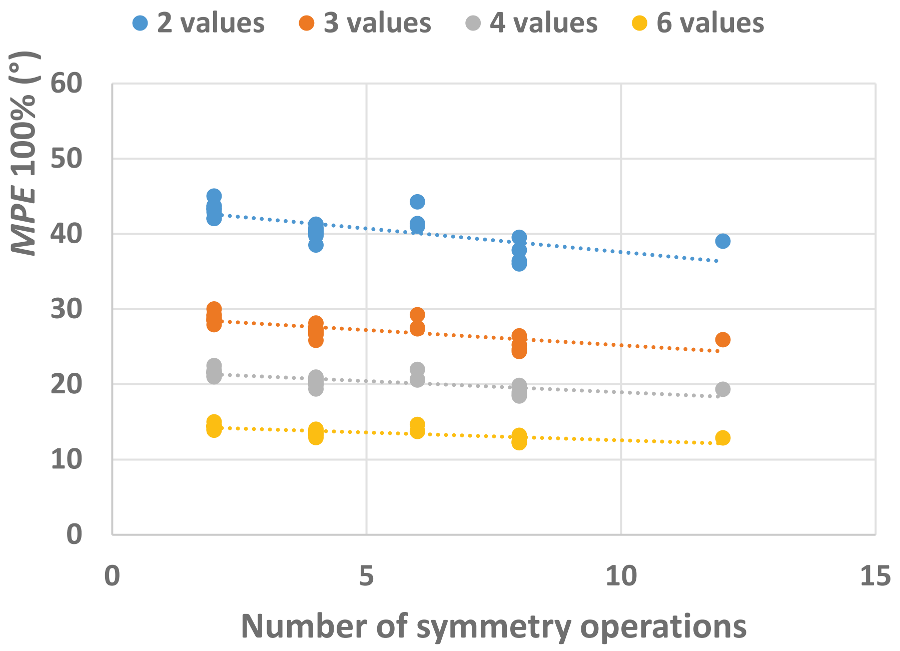

Figure S9 Mean phase errors of the maximum seed size, *i.e.* for $Perc_{seed}$=100% calculated for different sampling densities of the phase values as a function of the number of symmetry operations, calculated for all the large test structures used.

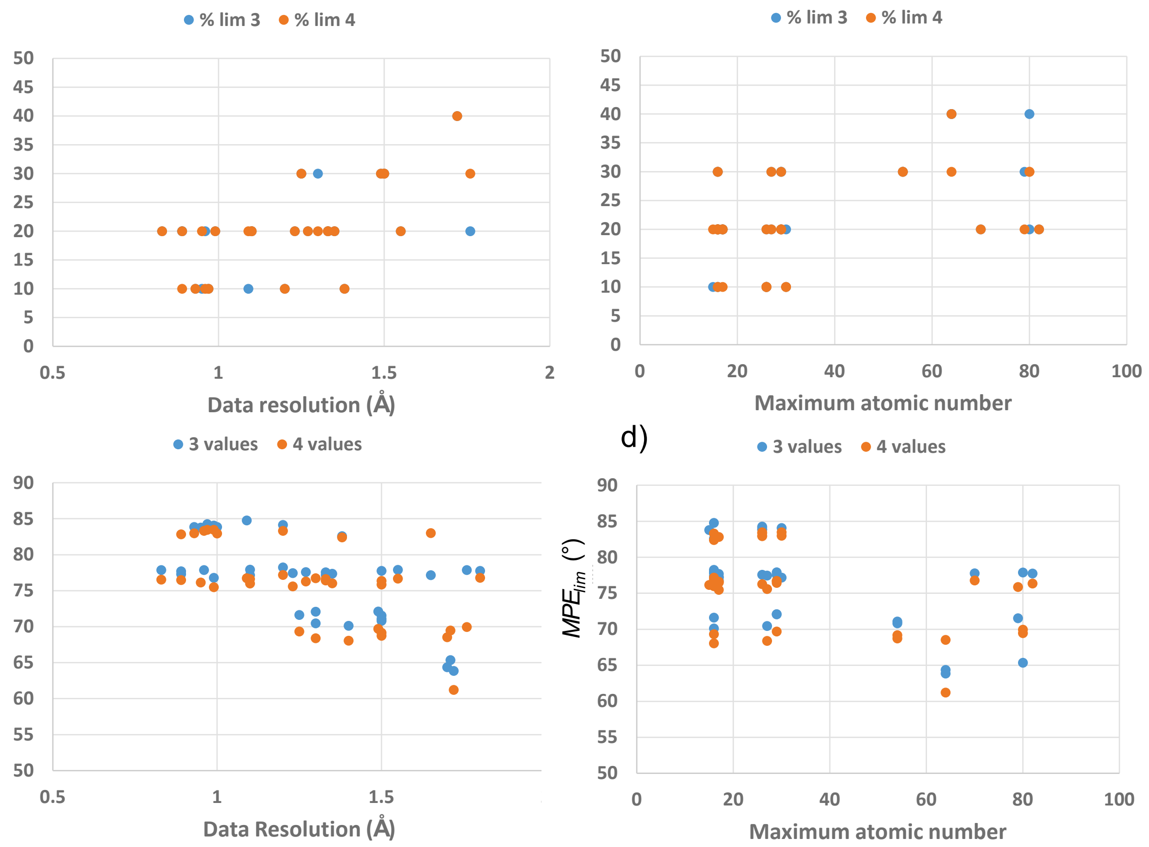

Figure S10 Minimum percentage of the number of seed reflections with respect to the total number of symmetry-independent reflections for which the phasing procedure leads to the correct structure solution ($Perc_{lim}$) (a, b) and maximum *MPE* for which the phasing procedure leads to the correct structure solution (MPE_{lim}) (c, d) obtained by using 3 (blue circles) and 4 (orange circles) sampling points for phase values, plotted as a function of the data resolution (a, c) and the atomic number of the heaviest atomic species present in the crystal (b, d), for all the large test structures used.

S6. Tests on powder data

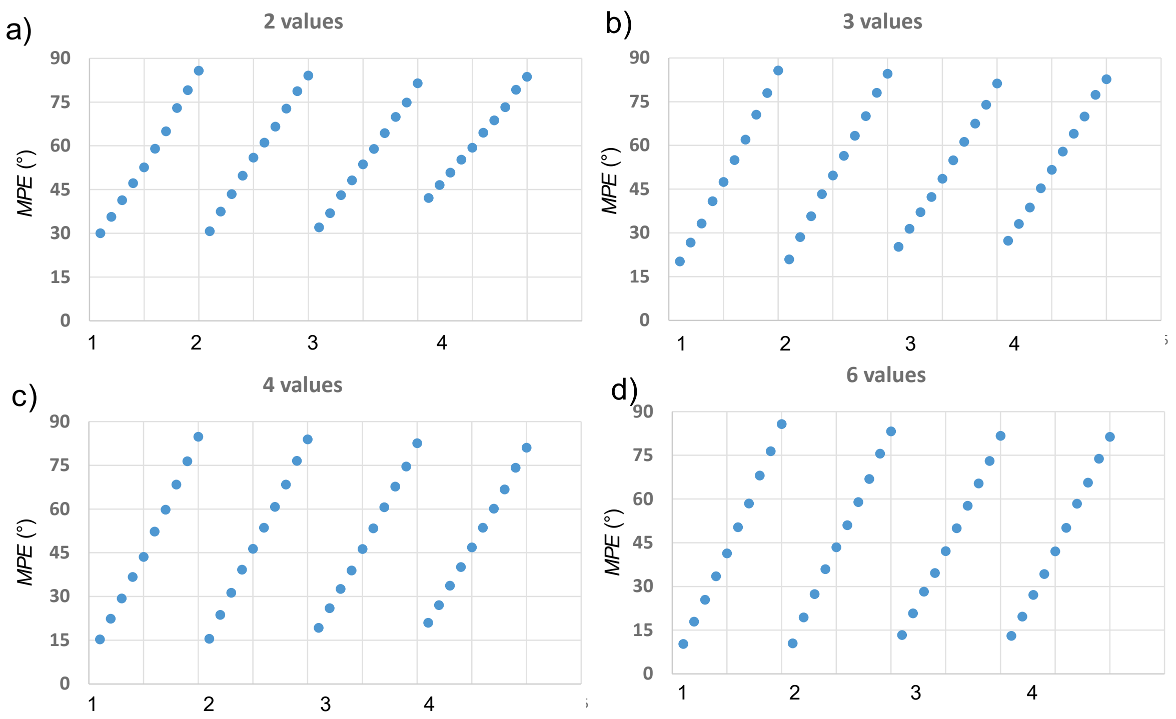

Figure S11 Mean phase errors obtained by using 2 (a), 3 (b), 4 (c) and 6 (d) sampling points for the structures from X-ray powder diffraction data.

S7. Key variables for the selection of reflections

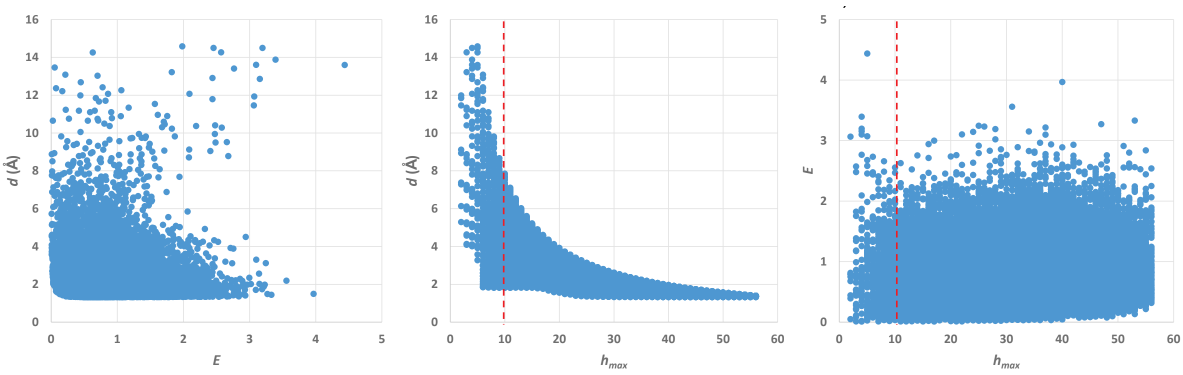

Figure S12 Scatter plots among three variables related to the 24111 symmetry-independent reflections of the protein with PDB code 193L: resolution (d), maximum value of the Miller index (h_{max}) and normalized structure factor (E). The effect of a hypothetical selection based on the value h_{max}=10 is shown by the red dashed line.
